# Influence of light at night on allergic diseases: a systematic review and meta-analysis

**DOI:** 10.1186/s12916-024-03291-5

**Published:** 2024-02-14

**Authors:** Andy Deprato, Robert Maidstone, Anna Palomar Cros, Ana Adan, Prasun Haldar, Barbara N. Harding, Paige Lacy, Lyle Melenka, Saibal Moitra, José Francisco Navarro, Manolis Kogevinas, Hannah J Durrington, Subhabrata Moitra

**Affiliations:** 1 Alberta Respiratory Centre and Division of Pulmonary Medicine, University of Alberta, Edmonton, AB Canada; 2 Michael G. DeGroote School of Medicine, McMaster University, Hamilton, ON Canada; 3 Division of Immunology, Immunity to Infection, and Respiratory Medicine, University of Manchester, Manchester, UK; 4 Non-Communicable Diseases and Environment Programme, Barcelona Institute for Global Health (ISGlobal), Barcelona, Spain; 5 Department of Experimental and Health Sciences, University of Pompeu Fabra (UPF), Barcelona, Spain; 6 Consortium for Biomedical Research in Epidemiology and Public Health (CIBERESP), Carlos III Institute of Health, Madrid, Spain; 7 Department of Clinical Psychology and Psychobiology, University of Barcelona, Barcelona, Spain; 8 Institute of Neurosciences, University of Barcelona, Barcelona, Spain; 9 Department of Medical Laboratory Technology, Supreme Institute of Management and Technology, Mankundu, India; 10 Synergy Respiratory and Cardiac Care, Sherwood Park, Alberta, Canada; 11 Department of Allergy and Immunology, Apollo Multispeciality Hospitals, Kolkata, India; 12 Department of Psychobiology and Methodology of Behavioural Sciences, University of Málaga, Málaga, Spain; 13 Hospital del Mar Medical Research Institute (IMIM), Barcelona, Spain; 14 Canadian VIGOUR Centre, Department of Medicine, University of Alberta, Edmonton, AB Canada

**Keywords:** Allergic rhinitis, Asthma, Chronotype, Shift work, Skin allergies

## Abstract

**Background:**

Allergic diseases impose a significant global disease burden, however, the influence of light at night exposure on these diseases in humans has not been comprehensively assessed. We aimed to summarize available evidence considering the association between light at night exposure and major allergic diseases through a systematic review and meta-analysis.

**Methods:**

We completed a search of six databases, two registries, and Google Scholar from inception until December 15, 2023, and included studies that investigated the influence of artificial light at night (ALAN, high vs. low exposure), chronotype (evening vs. morning chronotype), or shift work (night vs. day shift work) on allergic disease outcomes (asthma, allergic rhinitis, and skin allergies). We performed inverse-variance random-effects meta-analyses to examine the association between the exposures (ALAN exposure, chronotype, or shiftwork) and these allergic outcomes. Stratification analyses were conducted by exposure type, disease type, participant age, and geographical location along with sensitivity analyses to assess publication bias.

**Results:**

We included 12 publications in our review. We found that exposure to light at night was associated with higher odds of allergic diseases, with the strongest association observed for ALAN exposure (OR: 1.88; 95% CI: 1.04 to 3.39), followed by evening chronotype (OR: 1.35; 95% CI: 0.98 to 1.87) and exposure to night shift work (OR: 1.33; 95% CI: 1.06 to 1.67). When analyses were stratified by disease types, light at night exposure was significantly associated with asthma (OR: 1.62; 95% CI: 1.19 to 2.20), allergic rhinitis (OR: 1.89; 95% CI: 1.60 to 2.24), and skin allergies (OR: 1.11; 95% CI: 1.09 to 1.91). We also found that the association between light at night exposure and allergic diseases was more profound in youth (OR: 1.63; 95% CI: 1.07 to 2.48) than adults (OR: 1.30; 95% CI: 1.03 to 1.63). Additionally, we observed significant geographical variations in the association between light at night exposure and allergic diseases.

**Conclusions:**

Light at night exposure was associated with a higher prevalence of allergic diseases, both in youth and adults. More long-term epidemiological and mechanistic research is required to understand the possible interactions between light at night and allergic diseases.

**Supplementary Information:**

The online version contains supplementary material available at 10.1186/s12916-024-03291-5.

## Background

Almost all human biological functions are synchronized with an endogenous circadian timing system which is further influenced by several exogenous cues, known as “zeitgebers.” Of all known zeitgebers with the potential to regulate our biological rhythms, the light/dark cycle has the strongest influence [1, 2]. Humans are diurnal animals, i.e., we remain active primarily during the daytime and transition to dormancy at night; however, the discovery of electricity and the subsequent invention of artificial light has significantly extended our activity period into the night. These altered activity patterns have facilitated the rise of the evening chronotype, or “night owls,” where individuals are increasingly preferring to remain active mostly at night. Additionally, industrialization movements capitalized on artificial light with the conceptualization of night shift work, as the global economy started to require 24-h production to meet growing societal demands [3, 4]. Night shift work and evening chronotype, the two major risk factors for circadian misalignment, are known to be associated with sleep disruption and increased risk of several chronic diseases including metabolic and cardiovascular diseases, mental disorders, and cancer [5–9]. Despite this, a significant proportion of the population is active during the evening and overnight because of more nighttime activities, and nearly one-fifth of the working population in first-world countries work permanent or rotating night shifts [10].

**Fig. 1:**
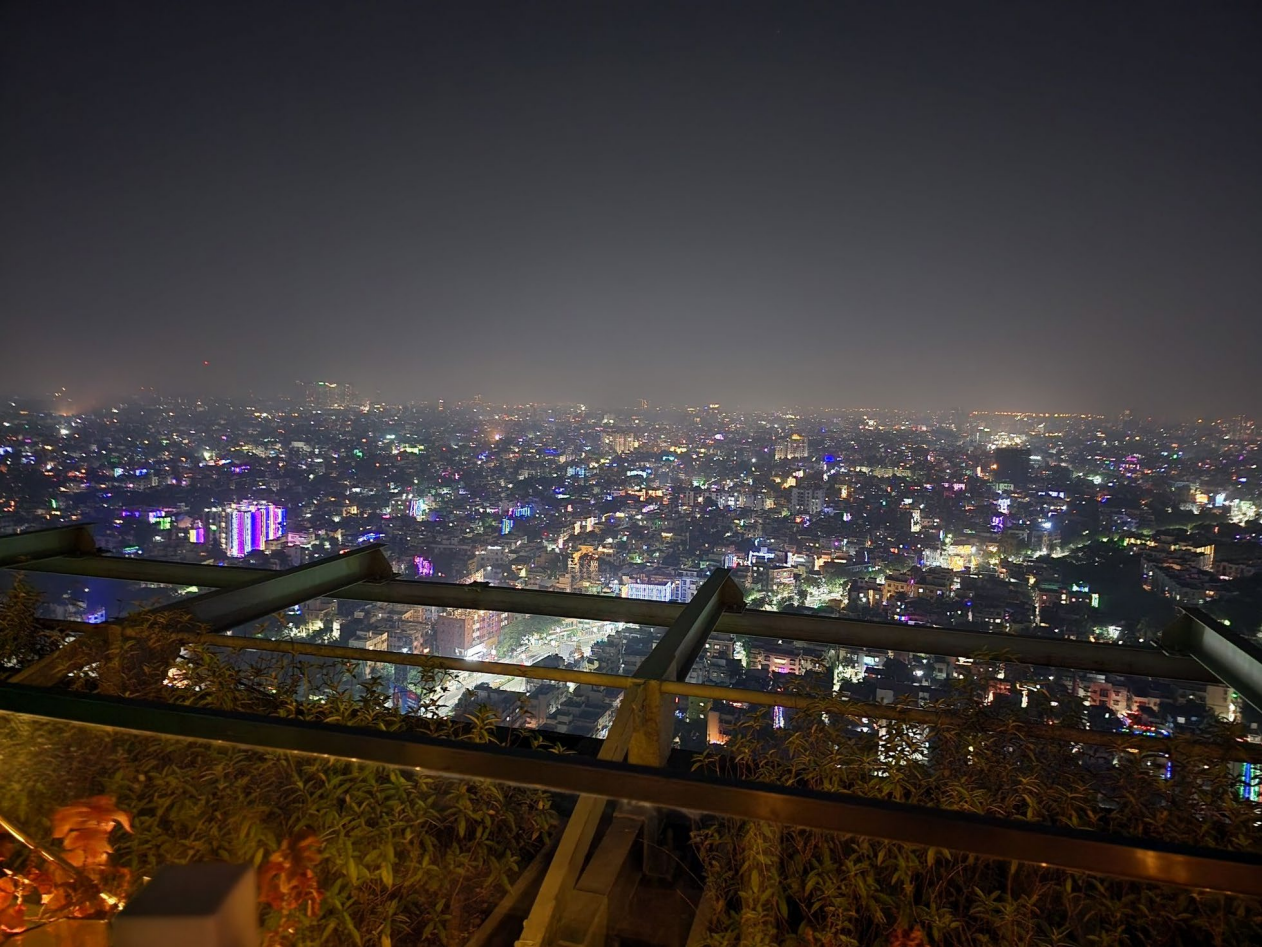
Street-level haze at night from light pollution in Kolkata, India on October 22, 2022. (Picture courtesy of Professor Bhramar Mukherjee, University of Michigan, USA)

In addition to the evening chronotype and night shift work, environmental light pollution, commonly known as artificial light at night (ALAN), has played a major role in disrupting the nocturnal environment and driving a large proportion of the global population towards circadian misalignment, particularly in cities and large urban centers [11]. Given global urbanization trends and the spread of technological infrastructure, more than 80% of the world’s population is estimated to be directly affected by outdoor ALAN. Nighttime skies are becoming increasingly polluted by excessive, misdirected, or obtrusive artificial light from several (mostly outdoor) sources, including residential or industrial areas, streetlights, and the reflection of this very light from the atmosphere (Fig. 1) [11–14]. For several decades, sodium vapor, metal halide, and fluorescent lamps were predominately used for outdoor lighting. However, due to lower production and maintenance costs, “white” light-emitting diode (LED) lamp use has increased exponentially, particularly in low-middle-income countries, consequently driving an increase in the blue light spectrum emitted by such light pollution [15]. Concurrently, indoor ALAN exposure at any hour continues to increase through lighting and personal devices, such as televisions, computer screens, and cell phones, all of which primarily emit blue-white light [16]. This increased blue-white light exposure is especially problematic due to its substantially greater ability to suppress melatonin secretion and circadian function compared to other wavelengths [17]. ALAN exposure has also been linked to chronic diseases and significantly influences metabolism, sleep, cancer, obesity, and mental health [18–23]. Further, these concerns also extend to those with evening chronotypes and night shift workers as they are also exposed to these light sources. In summary, ALAN imposes significant effects on circadian rhythmicity, which may lead to adverse effects on human health.

It is now well understood that ALAN can disrupt the rhythmicity of circadian genes through a Ca^2+^-dependent pathway [24]. Circadian genes play important roles in regulating the cascade of inflammatory gene expression in allergic diseases and ALAN-induced disruption of these genes may trigger proinflammatory responses, such as allergic reactions. Apart from direct control, several studies have shown that allergic diseases also have circadian rhythmicity [25–29]; for example, asthma and other allergic diseases such as allergic rhinitis and atopic dermatitis are known to have marked variations in symptoms at different times of the day due to underlying rhythmic inflammatory pathways [30–34]. Allergic diseases, including asthma, are very common chronic inflammatory diseases, affecting at least 8 to 10% of the global population [35]. Although several recent reports have suggested that an imbalance of light/dark homeostasis may be linked to allergy and asthma outcomes, there has not been a systematic analysis of the epidemiological evidence on this topic which could potentially bring attention to the hazards of light at night exposure. Given the availability of such epidemiological evidence, we performed a systematic review and meta-analysis to provide a comprehensive assessment of the associations between light at night exposure and allergic diseases, which could potentially provide valuable information for further studies and prevention strategies in this area.

## Methods

### Search strategy and study selection

This study was designed and conducted according to the Preferred Reporting Items for Systematic Reviews and Meta-Analyses (PRISMA) guidelines [36]. The protocol for this review, which includes further rationale and methodological details, was previously published on July 12, 2022 [37]. Since our review considers only published and publicly accessible information, it did not require formal ethics approval.

Eligibility criteria for included studies were guided by the population, exposure, comparison, outcome, and study design (PECOS) framework [36]. Studies that considered any human population with direct assessment of ALAN (indoor or outdoor ALAN) or indirect assessment of ALAN (evening chronotype or night shift work) were eligible for inclusion. Included studies must have compared the prevalence of allergic diseases (asthma, allergic rhinitis, and skin allergies) in high versus low (e.g., evening vs. morning chronotype) or yes versus no (night vs. day shift work) exposure groups. In the case of multiple levels of exposure in a study (ALAN), the highest versus lowest exposure approach was taken to compare outcomes in only the highest and lowest exposure groups. We included any primary studies (except case studies) published in English (or able to be translated) in peer-reviewed journals. We did not limit studies by publication country or date. If multiple studies were published for a given investigation, we only included the most recent or complete study as the parent study.

One author with library search experience (AD) developed and conducted a comprehensive search of six electronic databases, two registries, and Google Scholar from inception until December 2, 2022, through the University of Alberta library services. No filters or limiters were applied to any library searches and complete search strategies are included in the additional file (Additional file 1: Table S1). An updated search to identify studies published after the initial search was completed on December 15, 2023, through the McMaster University Health Sciences Library. Additional hand searches, bibliographic searches, and contact with study authors were also completed to identify any further studies. Search results were uploaded to the systematic review management software Covidence (Melbourne, VIC, Australia), where duplicate records were removed. One reviewer (first author) completed the initial title and abstract screening, and two reviewers (first and last author) independently assessed all remaining full texts for inclusion, with disagreements resolved through consensus or a third reviewer (HD). Content experts then reviewed and finalized the list of included studies.

### Data analyses

Two reviewers (first and last author) independently collected data from all included studies into Google Sheets (Google, Mountain View, CA, USA). Extracted data items included study and population characteristics, exposures (indoor or outdoor ALAN exposure, chronotype, or shift work), comparators, and outcomes. Content experts independently verified all extracted data. We performed meta-analyses to examine the association between light at night exposure (outdoor ALAN exposure, chronotype, or shift work) and all the allergic outcomes of interest in our review (asthma, allergic rhinitis, and skin allergies). Exposure-specific and outcome-specific meta-analyses were the main outcomes considered in this review, which were further explored through additional stratification analyses. For studies reporting more than one allergic outcome, we took the pooled estimate of those outcomes for our analyses. We estimated the overall association between light at night exposure and allergic outcomes by calculating the natural logarithms of odds ratios (OR) and corresponding standard errors (SE) for the pooled estimate from each study and applied an inverse-variance random-effects model to create the corresponding forest plot [38]. As many studies did not report adjusted ORs or reported other types of estimates such as prevalence ratios (PRs) or relative risks (RRs), we could not assess adjusted ORs. The ORs used for these analyses were either extracted from included studies or calculated from presented data; or if estimates were reported differently, such as through PRs or RRs. Heterogeneity was assessed by *I*^2^ statistics [39] and reported as low, moderate, and high if the *I*^2^ values were quantified as < 50%, 50% to 75%, and > 75%, respectively [40].

We stratified the meta-analyses by (i) each exposure and each outcome (e.g., outdoor ALAN and asthma), (ii) age group (youth and adults), and (iii) continent. Meta-analyses were completed for each outcome through Review Manager (RevMan) version 5.4 (Cochrane, London, UK). Significance was defined as two-sided *p* < 0.05.

### Quality assessment

Two reviewers (first and last author) independently assessed the risk of bias of included studies at the individual study level using the Risk of Bias In Non-randomized Studies – of Exposures (ROBINS-E) tool [41]. The Grading of Recommendations, Assessment, Development, and Evaluation (GRADE) framework was independently completed by two reviewers to assess the quality of evidence across included studies for each main outcome according to defined GRADE criteria [42]. We constructed funnel plots to assess the asymmetry of study results followed by Egger’s test [43] and Begg’s test [44] to check for publication bias in main outcomes. A *p*-value < 0.05 indicated significant publication bias.

## Results

### Search results and study characteristics

**Fig. 2:**
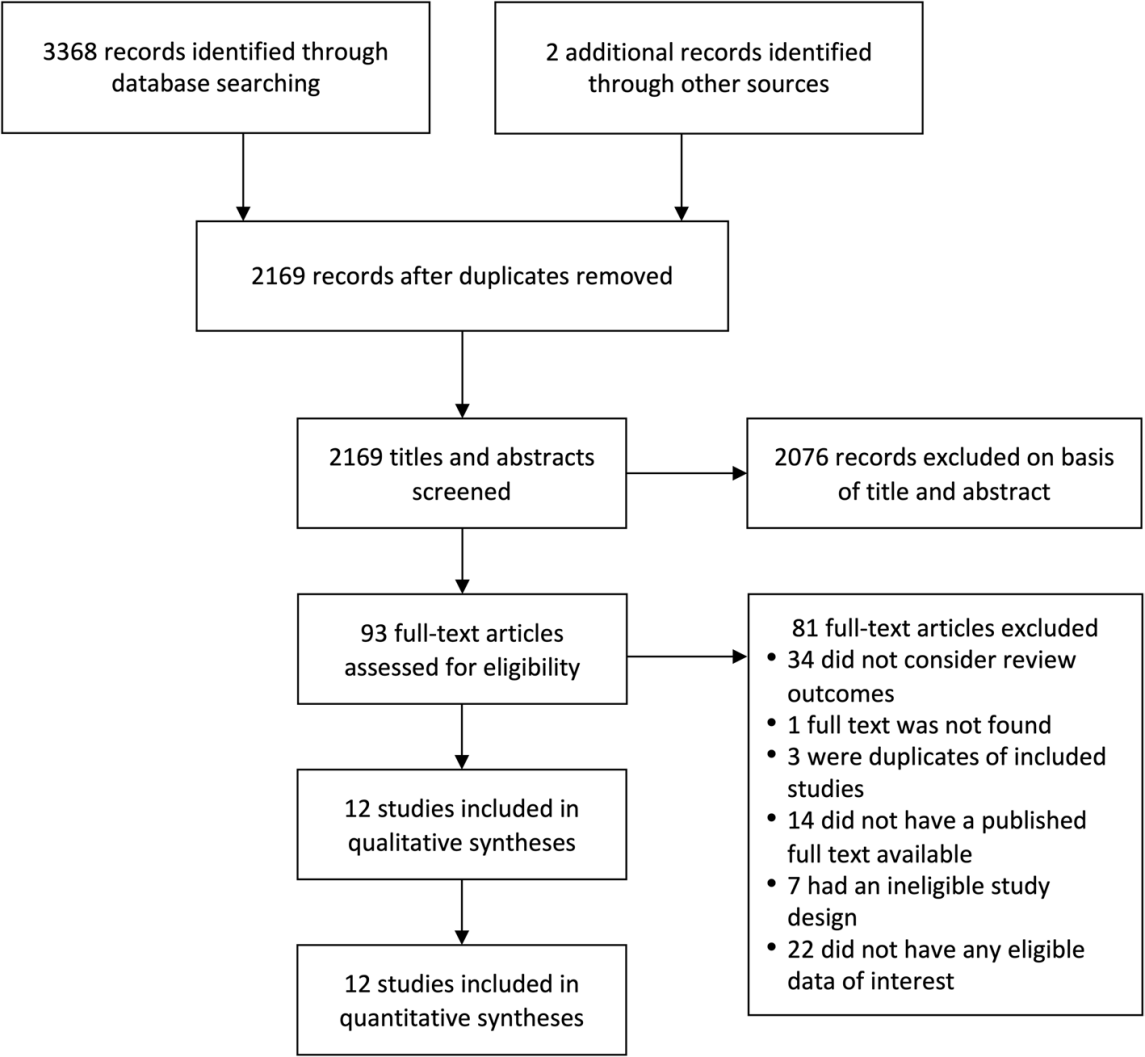
Preferred Reporting Items for Systematic Reviews and Meta-Analyses (PRISMA) flow diagram of the selection process for eligible studies considering the influence of light at night exposure on allergic diseases

The details of the study selection are presented in Fig. 2. We identified 3368 publication records through initial database searching, where after removing duplicates (*n* = 1199) and those that did not match our study questions (*n* = 2076), we completed a full-text review of 93 studies. We excluded full texts that did not consider the outcomes of interest of this review (*n* = 34), did not have any eligible data of interest (*n* = 22), had an ineligible study design (*n* = 7), or did not have a published full text available (*n* = 14). After a thorough screening, we included 12 studies with a total population of 855,917 individuals (563,937 adults and 291,980 children and adolescents) for subsequent qualitative and quantitative analyses. The summary of the updated search is presented in Additional file 1: Table S2.

The characteristics of each study included in the meta-analysis are presented in Table 1. All studies were cross-sectional, of which, only one directly examined ALAN exposure [45], whereas most studies considered chronotype (*n* = 6) [46–51] and shift work (*n* = 5) [52–56]. We did not find any studies that included more than one exposure at a time (e.g., ALAN and shift work and ALAN and chronotype). Over half of the included studies were reported from Asia (*n* = 7) [45, 47, 49, 50, 53–55], while fewer reports came from Europe (*n* = 3) [46, 51, 56] and the Americas (*n* = 2) [48, 52]. Allergic diseases in children and adolescents were addressed in five studies, and while almost all studies reported asthma as the primary outcome, allergic rhinitis (*n* = 6) and skin allergies (*n* = 5) were much less commonly considered.

**Table 1:** Characteristics of included studies considering the influence of light at night exposure on allergic diseases

Study	Study design	Country	Population	Exposure(s) considered	Outcome(s) considered	Quality assessment
Basnet et al. (2018) [46]	Cross-sectional	Finland	6424 adults (FINRISK study 2012)	Chronotype	Asthma	Low risk of bias
Chen et al. (2022) [47]	Cross-sectional	China	10,409 primary school children (Shanghai Children’s Allergy Study)	Chronotype	Asthma, allergic rhinitis, and skin allergies	Some concerns for bias
Ferreira et al. (2022) [48]	Cross-sectional	Brazil	1457 adolescents	Chronotype	Asthma	Some concerns for bias
Fischer et al. (2001) [52]	Cross-sectional	Brazil	124 adult male printing company workers	Shift work	Asthma, allergic rhinitis, and skin allergies	High risk of bias
Haldar et al. (2020) [49]	Cross-sectional	India	1684 adolescents (PERFORMANCE study)	Chronotype	Asthma, allergic rhinitis, and skin allergies	Low risk of bias
Han and Chung (2020) [50]	Cross-sectional	South Korea	278,430 adolescents (Korea Youth Risk Behaviour Web-Based Survey)	Chronotype	Asthma	Low risk of bias
Huang et al. (2021) [53]	Cross-sectional	China	7411 automobile manufacture workers (Dongfeng-Tongji Cohort Study) and non-ferrous metal smelting workers (Hunan Chronic Disease Cohort Study)	Shift work	Skin allergies	Low risk of bias
Kim et al. (2020) [54]	Cross-sectional	South Korea	20,613 female nurses (Korea Nurses’ Health Study)	Shift work	Skin allergies	Some concerns for bias
Lu et al. (2019) [55]	Cross-sectional	The Philippines	630 call center and business outsourcing employees	Shift work	Skin allergies	High risk of bias
Maidstone et al. (2021) [56]	Cross-sectional	UK	502,540 adults (UK Biobank)	Shift work	Asthma	Low risk of bias
Merikanto et al. (2014) [51]	Cross-sectional	Finland	6089 adults (FINRISK study 2007)	Chronotype	Asthma and allergic rhinitis	Low risk of bias
Tang et al. (2022) [45]	Cross-sectional	China	20,106 college students	Outdoor artificial light at night	Asthma, allergic rhinitis, and skin allergies	Low risk of bias

Studies were listed in alphabetical order

### Light at night and allergic diseases

**Fig. 3:**
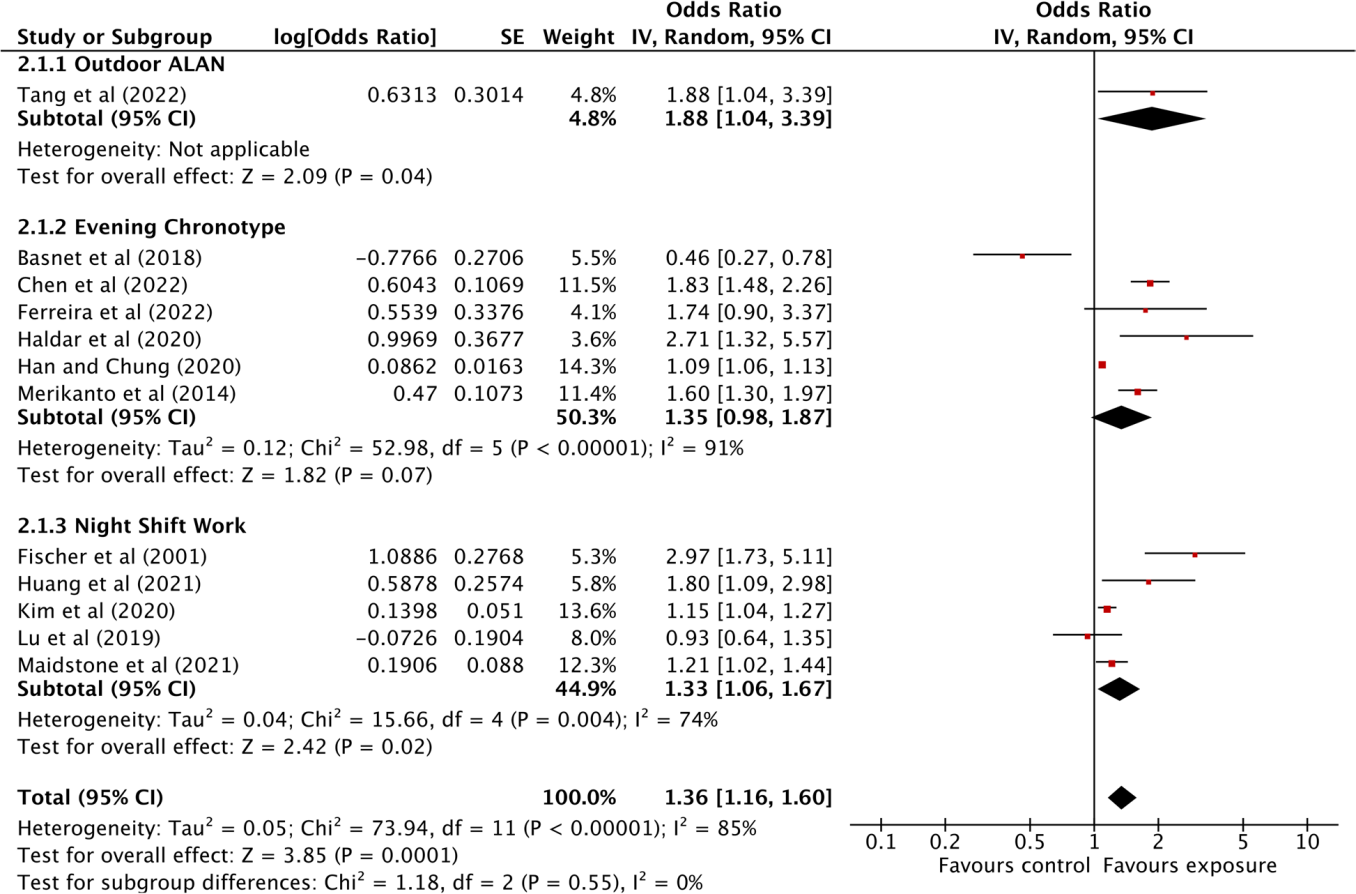
Association between light at night exposure and the odds of allergic diseases stratified by exposure type. Analyses were conducted with an inverse-variance random-effects model. Each square represents the reported odds ratios in each original study, and the size of the square represents the pooled weight that the study was given according to the sample size. The line through the square indicates the corresponding confidence interval (CI). Diamonds represent the overall effect in pooled studies

**Fig. 4:**
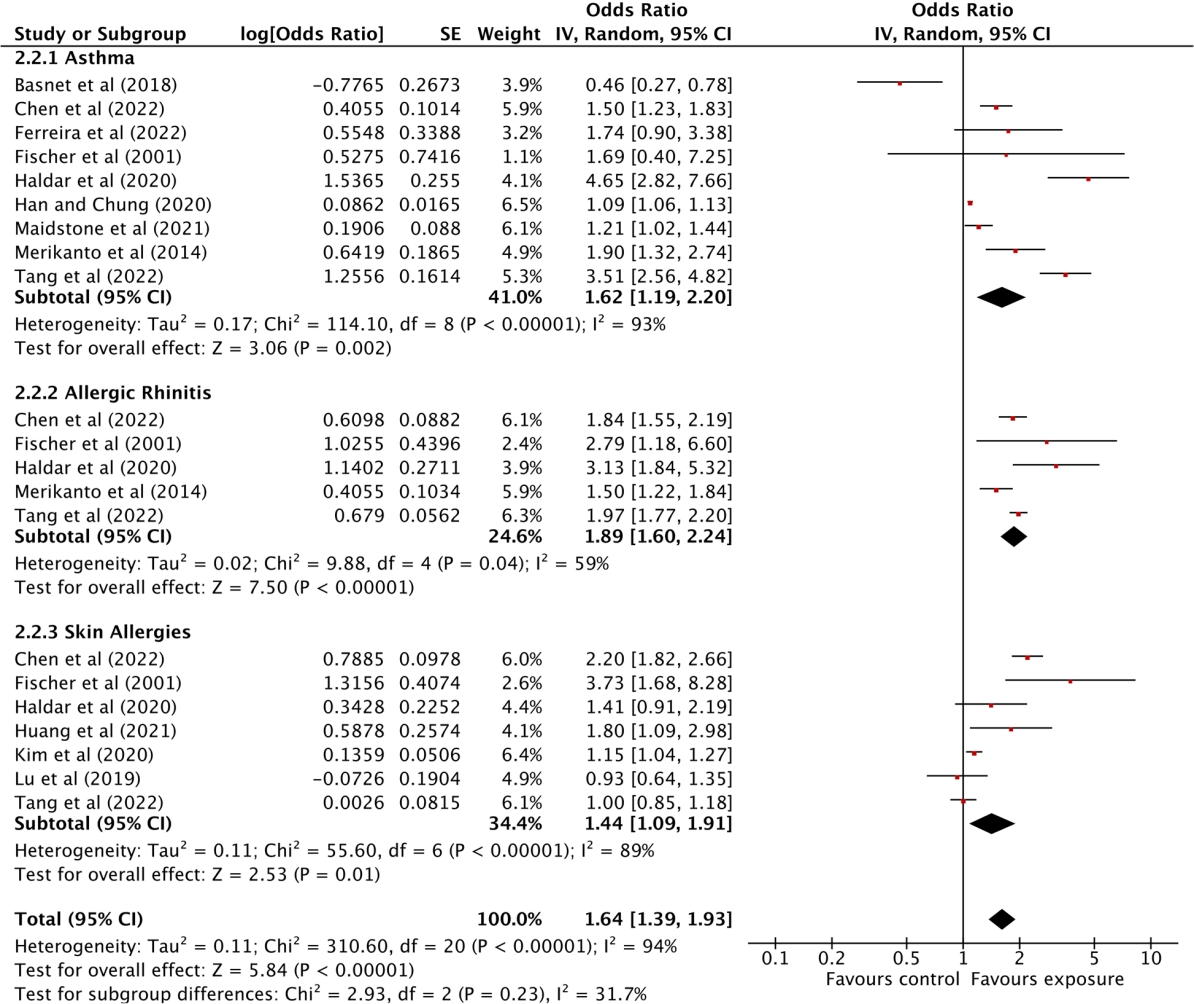
Association between light at night exposure and the odds of allergic diseases stratified by type of allergic disease. Analyses were conducted with an inverse-variance random-effects model. Each square represents the reported odds ratios in each original study, and the size of the square represents the pooled weight that study was given according to the sample size. The line through the square indicates the corresponding confidence interval (CI). Diamonds represent the overall effect in pooled studies

**Fig. 5:**
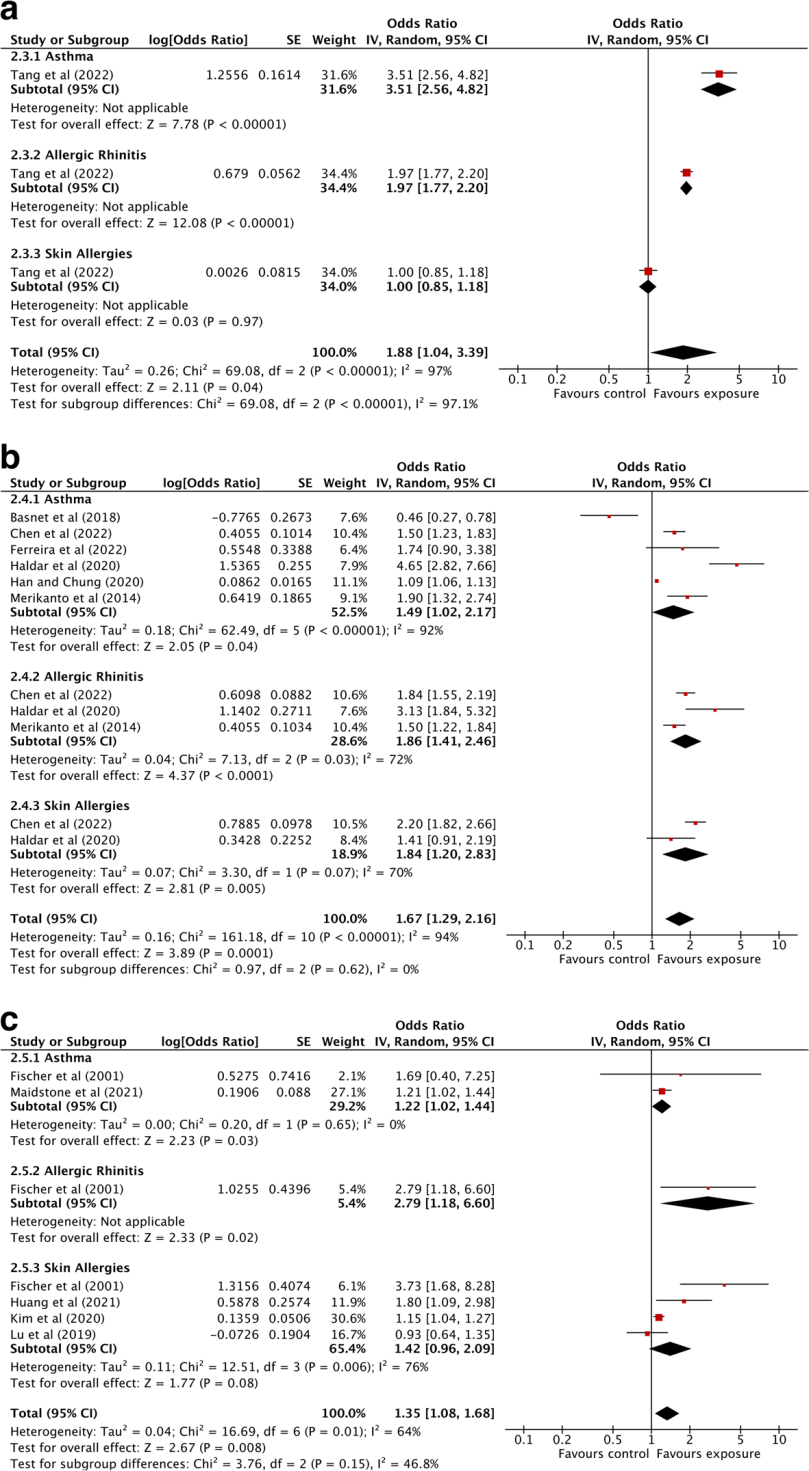
Association between light at night exposure and the odds of allergic diseases stratified by type of allergic disease for **a** artificial light at night exposure, **b** chronotype, and **c** shift work. Analyses were conducted with an inverse-variance random-effects model. Each square represents the reported odds ratios in each original study, and the size of the square represents the pooled weight that the study was given according to the sample size. The line through the square indicates the corresponding confidence interval (CI). Diamonds represent the overall effect in pooled studies

**Fig. 6:**
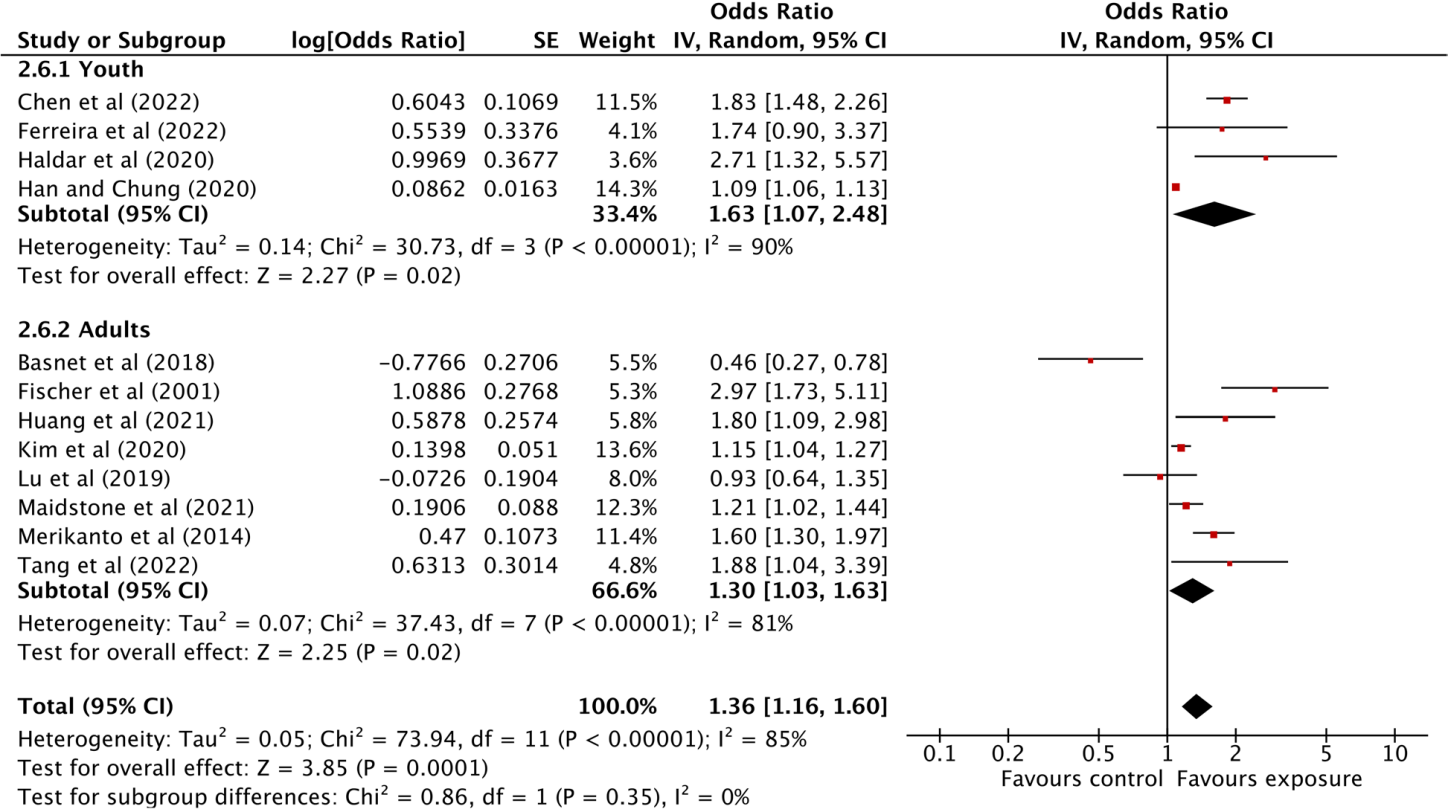
Association between light at night exposure and the odds of allergic diseases stratified by age group. Analyses were conducted with an inverse-variance random-effects model. Each square represents the reported odds ratios in each original study, and the size of the square represents the pooled weight that the study was given according to the sample size. The line through the square indicates the corresponding confidence interval (CI). Diamonds represent the overall effect in pooled studies

The associations between light at night exposure and any allergic diseases are summarized in Fig. 3. The only study examining outdoor ALAN exposure in relation to allergic diseases showed a significant association between outdoor ALAN exposure and allergic diseases (OR: 1.88; 95% CI: 1.04 to 3.39). However, having an evening chronotype was found to be marginally associated with higher odds of allergic diseases (OR: 1.35; 95% CI: 0.98 to 1.87) while those exposed to night shift work had significantly higher odds of having any allergic diseases (OR: 1.33; 95% CI: 1.06 to 1.67). Through our outcome-specific stratification analyses, we found that any means of exposure to light at night was significantly associated with higher odds of asthma (OR: 1.62; 95% CI: 1.19 to 2.20), allergic rhinitis (OR: 1.89; 95% CI: 1.60 to 2.24), and skin allergies (OR: 1.44; 95% CI: 1.09 to 1.91) (Fig. 4). In the subgroup analysis by each exposure and each outcome, we found that outdoor ALAN was significantly associated with asthma (OR: 3.51; 95% CI: 2.56 to 4.82) and allergic rhinitis (OR: 1.97; 95% CI: 1.77 to 2.20), but not skin allergies (OR: 1.00; 95% CI: 0.85 to 1.18) (Fig. 5a). However, due to only one study on outdoor ALAN, we could not estimate heterogeneity. Similarly, evening chronotype was significantly associated with all three outcomes, asthma (OR: 1.49; 95% CI: 1.02 to 2.17), allergic rhinitis (OR: 1.86; 95% CI: 1.41 to 2.46), and skin allergies (OR: 1.84; 95% CI: 1.20 to 2.83) (Fig. 5b). Night shift work was significantly associated with asthma (OR: 1.22; 95% CI: 1.02 to 1.44) and allergic rhinitis (OR: 2.79; 95% CI: 1.18 to 6.60), but not with skin allergies (OR: 1.42; 95% CI: 0.96 to 2.09) (Fig. 5c). We also found that the associations between light at night exposure and any allergic diseases were higher among youths (OR: 1.63; 95% CI: 1.07 to 2.48) compared to adults (OR: 1.30; 95% CI: 1.03 to 1.63) in our age-specific stratification analyses (Fig. 6).

**Fig. 7:**
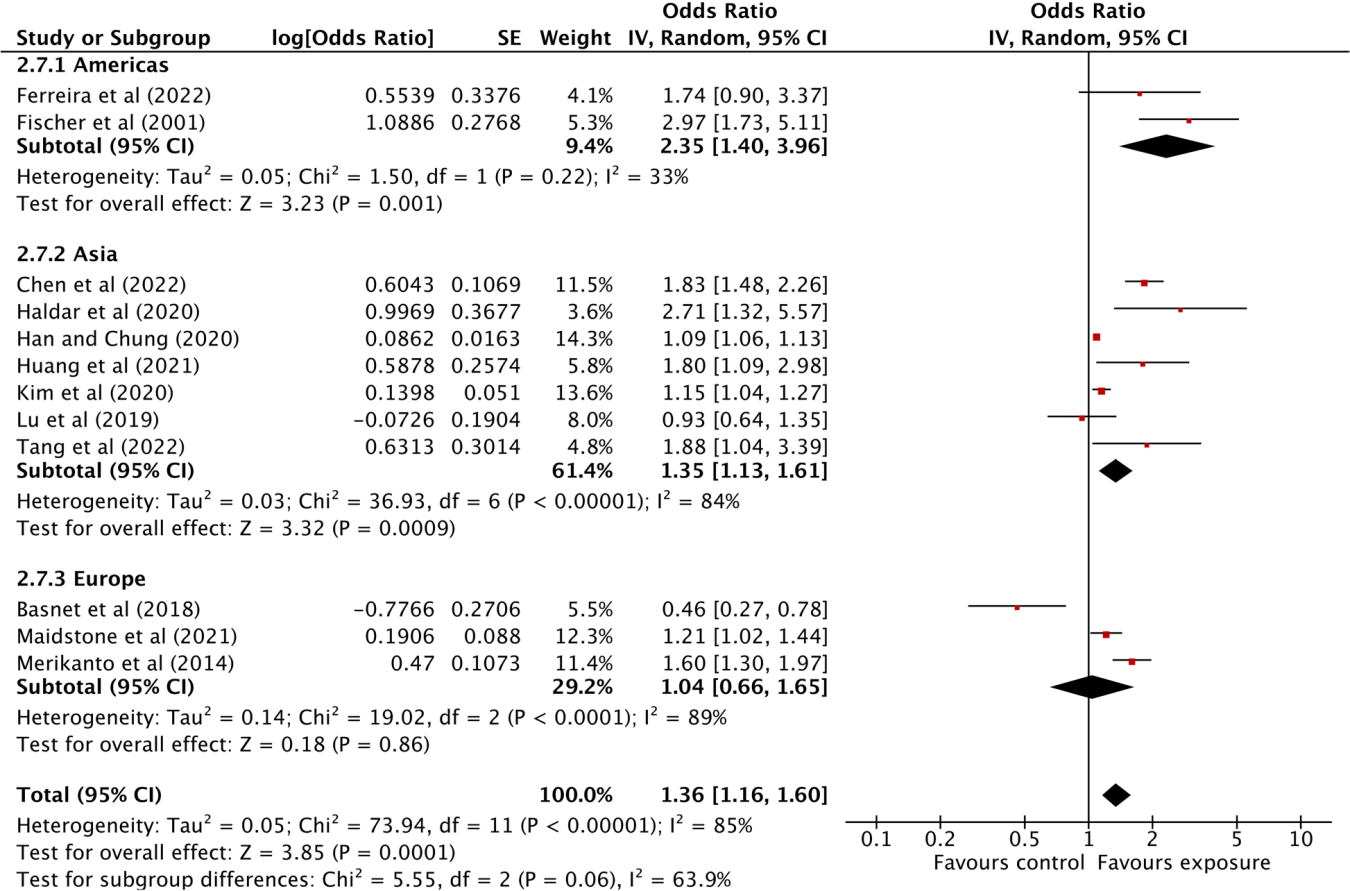
Association between light at night exposure and the odds of allergic diseases stratified by continent. Analyses were conducted with an inverse-variance random-effects model. Each square represents the reported odds ratios in each original study, and the size of the square represents the pooled weight that the study was given according to the sample size. The line through the square indicates the corresponding confidence interval (CI). Diamonds represent the overall effect in pooled studies

Studies published in the Americas showed a significant positive association between light at night exposure and allergic diseases (OR: 2.35; 95% CI: 1.40 to 3.96) with low heterogeneity (*I*^2^: 33%), while Asian studies showed relatively less strong associations between light at night exposure and any allergic diseases, although these studies were significantly heterogeneous (OR: 1.35; 95% CI: 1.13 to 1.61; *I*^2^: 84%). However, the pooled estimate of the European studies did not exhibit any significant associations between light at night exposure and allergic diseases (OR: 1.04; 95% CI: 0.66 to 1.65; *I*^2^: 89%) (Fig. 7). A summary of all analyses is presented in Additional file 1: Table S3.

### Quality assessment

After visually inspecting the funnel plots and Egger’s and Begg’s tests in the quality assessment for each exposure and allergic outcome, we found very little publication bias among studies assessing the association between evening chronotype and any allergic conditions (Egger’s and Begg’s test *p*-values were 0.31 and 0.85, respectively). Similarly, very little bias was observed among studies investigating night shift work and allergic outcomes (Egger’s and Begg’s test *p*-values were 0.27 and 0.14, respectively). However, we could not assess bias for outdoor ALAN and allergic outcomes for limited studies (*n* = 1) (Additional file 1: Fig. S1). Similarly, in the analysis for any ALAN exposure and each of the allergic outcomes, we observed little bias among studies (Additional file 1: Fig. S2).

Overall, a majority (7 out of 12) of the included studies had low risk, three studies had some concerns, and only 2 studies were of high risk of bias according to the ROBINS-E tool for bias assessment (Additional file 1: Fig. S3). One-third of the studies had some bias due to confounding, and only one study (8%) had bias arising from the measurement of the outcome. However, there were no major biases in terms of exposure assessment, selection of participants, post-exposure interventions, missing data, and selection of the reported results (items D2-D5 and D7 of the ROBINS-E tool). As all studies were observational, the quality of overall evidence was very low to moderate as per the GRADE criteria, however, there were no significant concerns identified in the certainty assessments (Additional file 1: Table S4).

## Discussion

### Study findings

This meta-analysis synthesized epidemiological evidence of the association between exposure to light at night and allergic diseases. Both outdoor ALAN and night shift work were found to be significantly associated with higher odds of allergic diseases while we found a borderline significant association between evening chronotype and allergic diseases. Light at night exposure had remarkable associations with all major allergic diseases such as asthma, allergic rhinitis, and skin allergies, and the associations were stronger among youth than adults. While studies from the Americas and Asia strongly supported a positive association between light at night exposure and allergic diseases, European studies provided mixed evidence.

The associations identified in the meta-analysis between circadian misalignment and allergic diseases are broadly consistent with previous narrative reviews Circadian misalignment could take place due to phase delays as a result of shifting towards night, particularly because of a disrupted melatonin cycle [57–60]. Although the concept of “nocturnal asthma” was proposed more than 50 years ago [61], several recent reports have come up with more supportive evidence of the involvement of circadian clocks in nocturnal symptoms in asthma, primarily from laboratory investigations [31, 51, 62]. Apart from the only study on outdoor ALAN, studies investigating associations between evening chronotype, and allergic diseases found a borderline significant association (*p* = 0.07). This could partly be because of the mixed population, i.e., youths and adults, whereas in occupational studies on shift work, a stronger association between light at night and allergic diseases was observed [52–56]. Additionally, the stronger association between light at night and allergic diseases in youths indicates that circadian misalignment might have a stronger influence on biological function in this population compared to adults. This might be explained by younger children typically being more morning-oriented than adults [63–65], where a sudden change of greater magnitude in phase transition may lead to greater health consequences among this younger population. Lastly, studies have found that geographical location plays an important role in determining the circadian preference of an individual, mainly because of natural daylight and cultural reasons [66, 67]. Moreover, the prevalence of allergic diseases differs across continents [68], all of which might explain the intercontinental variation of the relationships between light at night exposure and allergic diseases we observed. Nevertheless, the number of studies from Asia was much higher than studies published from other continents, which might also explain the variability in the associations.

The studies analyzed in this meta-analysis were heterogeneous, partly because of the nature of data integration for the analysis. First, we calculated unadjusted ORs for some studies while for other studies, we had to consider adjusted ORs (minimally adjusted models). Therefore, the possibility of confounding effects of major determinants such as age and sex could not be reflected in unadjusted ORs. Further, several included studies had very large sample sizes which could inherently influence statistical calculations towards greater heterogeneity. We found little publication bias for light at night and allergic disease studies, mostly because there were comparatively fewer studies with negative or null associations, presumably, because of the uneven sample size of studies included in this analysis. However, a similar result from our sensitivity analysis supports the notion that our findings were not driven by publication bias.

### Clinical and public health implications

The prevalence of allergic diseases, mainly respiratory allergies such as asthma and allergic rhinitis, is increasing globally at a rapid rate and is a major health concern for children and adolescents. The management of these diseases is often behavioral, such as changes in lifestyle and diet. Younger generations have become mostly night-oriented due to lifestyle or work-related conditions and such phase delays are often detrimental to health. While experimental studies have presented a link between light at night mediated circadian disruption and immunological manifestations of allergic diseases [27, 69–71], simple physical modifications, such as changing indoor lighting to amber and switching computer or mobile screens to night mode could help attenuate this detrimental relationship. Investigations on the circadian rhythmicity of allergic diseases also vouch for chronotherapy in allergic diseases, i.e., timing medication based on the timing of symptoms [72, 73]. This might present a more favorable way of managing allergic diseases to some, while potentially also reducing the dosage and over-reliance of particular medications by patients although we must be aware that the causal relationships between circadian dysregulation and allergic diseases are still to be established. It must be remembered that childhood allergies can have serious health consequences at a later stage in life if they remain untreated. Therefore, parents must be vigilant in ensuring their younger children do not develop overly evening-oriented lifestyles to minimize the possibility of over-exposure to light at night. From an occupational perspective, certain professions require night shift work (e.g., healthcare, first response, law enforcement) which could predispose potentially susceptible individuals to allergy-aggravating light at night exposure. Considering appropriate physical, policy, or ergonomic controls (e.g., blue-light filtering glasses, decreased use of blue-white lighting, or reducing night shift work for individuals with severe allergies) is critical in ensuring the well-being of individuals in these occupations.

### Potential mechanisms

The circadian clock is an important regulator of allergic reactions (29). Reports also suggest that most allergic diseases follow circadian rhythmicity as some of the major cellular functions in allergies such as mast cell activation or epithelial barrier function are dependent on circadian timing [26–28]. Although the physiological circadian variation in atopic conditions or change in airway caliber in asthma are directly controlled by central and peripheral biological clocks rather than external environmental cues such as light/dark cycles [74], little has been explored so far about the roles of external environmental influences on allergies and asthma. Two recent reports showed that the airway eosinophils, fractional exhaled nitric oxide, and volatile organic compounds in the exhaled breath of patients with asthma follow circadian rhythmicity [30, 75], although the underlying mechanism is still not well understood. Although some animal studies demonstrated that circadian rhythm disruption could lead to systemic allergic reactions such as allergic rhinitis in mice possibly by elevating Th_2_-like immune responses [76], human studies in this area have not evolved significantly. In one recent study among 51 male rotating shift workers from a car industry in Barcelona, Spain, investigators found that night shift was associated with disruption of multiple immune response pathways, which could link to an altered allergic response [77], As ALAN, night shift work, and eveningness are known to cause circadian disruption, a probable link between light at night exposure and allergic diseases could be mediated through circadian misalignment, which further connects to disruption in melatonin homeostasis. Although typically considered the sleep hormone, recent studies have focused on melatonin for its immunomodulatory role, particularly in asthma and allergic diseases, especially when the biological clock is dysregulated or disrupted [78]. However, the role of melatonin in asthma has remained unclear and evidence as to whether melatonin acts as a pro- or anti-inflammatory substance has been contradictory. More studies are required to understand the potential mechanisms of circadian misalignment in asthma and allergic rhinitis.

It has been demonstrated that the physiological and immunological functions of the skin follow circadian rhythmicity. For example, skin hydration and barrier function reach *zenith* during the daytime [34, 79], while circulating naïve T-cells and pro-inflammatory cytokines, such as interleukin (IL)-12 reach their peak at night in adults [80, 81]. Skin integrity such as hydration and defense mechanisms is further regulated by circadian *CLOCK* (Circadian Locomotor Output Cycles Kaput) and *BMAL1* (Basic Helix-Loop-Helix ARNT Like 1) genes [82]. Thus, a circadian phase disruption caused by ALAN, shift work, or eveningness could lead to irregular expression of these circadian genes, and as a result, the downstream functions of these genes are greatly affected, leading to clinical conditions such as atopic dermatitis [82]. Allergic skin conditions such as atopic dermatitis and eczema are also influenced by melatonin. Previous studies suggested that melatonin offers a protective role in eczema by reducing circulating immunoglobulin (Ig)-E antibodies [78]. In a double-blind randomized controlled study, children with atopic dermatitis who received melatonin supplementation had a significantly improved disease condition than those receiving a placebo [83]. Thus, melatonin suppression caused by exposure to light at night further facilitates skin allergies.

### Strengths and limitations

This is the first meta-analysis of population-based studies summarizing the associations between exposure to light at night and allergic diseases. We have included multiple studies among populations residing at various geological locations and with differently measured exposures to light at night. One of the major strengths of this review is that included studies used standard measures of light at night exposure, i.e., either by direct measurement of ALAN, by job category (day vs. night shift work), or by standardized questionnaires of morningness-eveningness. Secondly, studies were available on both youth and adult populations, which minimizes the possibility of specific age-dependent responses. Lastly, most studies had reliable moderate-to-large sample sizes and well-described statistical methodologies. One of the common weaknesses of these studies is that their study participants belonged to specifically selected groups of people of whom the exposure to light at night was examined, except for some large nationwide cohorts. Second, all studies included in this analysis were cross-sectional, thus a causal interpretation of study findings could not be made. Third, no included studies considered more than one exposure to light at night, and thus, we could not identify any synergistic role of multiple exposures on allergic diseases. Fourth, we found only one study on outdoor ALAN eligible for inclusion in this study [45], therefore, we could not compare it with other studies of similar exposure. Moreover, the said study did not have a reference group (group without potential exposure to outdoor ALAN); therefore, we could only compare the dose–response (high vs. low) relationship of outdoor ALAN and could not compare the net influence of outdoor ALAN on the allergic disease outcomes. This study focuses on outdoor ALAN and data was assessed from satellite image (750 m resolution), which could have a possible potential for exposure misclassification. We need more studies on an individual level and on indoor exposure (if participants have light-blocking mechanisms this outdoor ALAN may not enter participants’ bedrooms). Fifth, evening chronotype and night shift work were used as a proxy for ALAN exposure and might not be properly classified as ALAN exposure regarding exposure assessment. It should be remembered that there could be additional job-related exposures in shift work such as shift duration, shift rotation, work environment, job stress, and excessive workload, which might impart additional risks for allergic diseases; therefore, more direct evidence of outdoor ALAN and allergic diseases is needed. Sixth, we observed high heterogeneity in the included studies in regard to the estimates of the associations between the exposure and the disease of interest, which might be considered as a limitation of this study. This high heterogeneity could be due to multiple factors such as study participants (children, youths, or adults, different sexes, etc.), exposure types, duration and assessment, analytical models, and last but not least, geographical locations; however, despite grouping similar exposures and outcomes, the heterogeneity persisted, which indicates involvement of possible residual confounding in those studies. Additionally, as human studies linking circadian rhythms and allergic diseases are relatively fewer and mostly observational, it was not possible to perform more in-depth sensitivity analyses or dose–response analyses. Seventh, as all studies except one [45] did not have a quantitative assessment of exposure, we were unable to perform any dose–response meta-analysis. Lastly, allergic diseases are greatly influenced by several other social, environmental, and genetic determinants [84–92], which were not taken into account in most of the studies considered, and thus the possible involvement of any mediators or effect modifiers such as socioeconomic condition, air pollution, or parental allergy could not be confirmed in this analysis.

## Conclusions

In conclusion, this meta-analysis shows overall positive associations between light at night exposure and allergic diseases, especially for asthma and allergic rhinitis. The association was stronger among youth than adults. Although the possible mechanisms of such associations have not been established yet, it is assumed from experimental studies that phase delays may disrupt melatonin rhythms, which may influence downstream immunological pathways. More studies, preferably interventional in design, are warranted to extend this research to reduce or protect against light at night exposure and assess whether these findings can be translated into changes in clinical practice to improve care and treatment for allergic diseases.

### Supplementary Information

**Additional file 1: Table S1.** Comprehensive strategy of the initial search to identify studies considering the influence of light at night exposure on allergic diseases. **Table S2.** Comprehensive strategy of the updated search to identify studies considering the influence of light at night exposure on allergic diseases. **Table S3.** Summary of meta-analyses considering the association between light at night exposure and the odds of allergic diseases. **Fig. S1.** Funnel plot of the exposure-specific meta-analysis for the association between light at night exposure and the odds of allergic diseases. **Fig. S2.** Funnel plot of the outcome-specific meta-analysis for the association between light at night exposure and the odds of allergic diseases. **Fig. S3.** Risk of Bias In Non-randomized Studies – of Exposures (ROBINS-E) quality assessment of included studies considering the influence of light at night exposure on allergic diseases. **Table S4.** Grading of Recommendations, Assessment, Development, and Evaluation (GRADE) criteria evidence table of meta-analyses considering the influence of light at night exposure on the odds of allergic diseases.

## Data Availability

We did not create any new data for this manuscript. All data associated with the manuscript were previously published and can be accessed from the respective journals.

## Abbreviations

ALAN: Artificial light at night
*BMAL1*: Basic Helix-Loop-Helix ARNT Like 1
CI: Confidence interval
*CLOCK*: Circadian Locomotor Output Cycles Kaput
GRADE: Grading of Recommendations, Assessment, Development, and Evaluation
IgE: Immunoglobulin E
LED: Light-emitting diode
OR: Odds ratio
PECOS: Population, exposure, comparison, outcome, and study design framework
PR: Prevalence ratio
PRISMA: Preferred Reporting Items for Systematic Reviews and Meta-Analyses
ROBINS-E: Risk of Bias In Non-randomized Studies – of Exposures
RR: Relative risk
SE: Standard error

## References

[1] Refinetti R (2016). Circadian Physiology.

[2] Allada R, Bass J (2021). Circadian mechanisms in medicine. N Engl J Med.

[3] Lunn RM, Blask DE, Coogan AN, Figueiro MG, Gorman MR, Hall JE (2017). Health consequences of electric lighting practices in the modern world: a report on the National Toxicology Program's workshop on shift work at night, artificial light at night, and circadian disruption. Sci Total Environ.

[4] Seto KC, Golden JS, Alberti M, Turner BL (2017). Sustainability in an urbanizing planet. Proc Natl Acad Sci U S A.

[5] Wegrzyn LR, Tamimi RM, Rosner BA, Brown SB, Stevens RG, Eliassen AH (2017). Rotating night-shift work and the risk of breast cancer in the nurses' health studies. Am J Epidemiol.

[6] Vetter C, Dashti HS, Lane JM, Anderson SG, Schernhammer ES, Rutter MK (2018). night shift work, genetic risk, and type 2 diabetes in the UK biobank. Diabetes Care.

[7] Mason IC, Qian J, Adler GK, Scheer F (2020). Impact of circadian disruption on glucose metabolism: implications for type 2 diabetes. Diabetologia.

[8] Jankowiak S, Backe E, Liebers F, Schulz A, Hegewald J, Garthus-Niegel S (2016). Current and cumulative night shift work and subclinical atherosclerosis: results of the Gutenberg Health Study. Int Arch Occup Environ Health.

[9] Chellappa SL, Vujovic N, Williams JS, Scheer F (2019). Impact of circadian disruption on cardiovascular function and disease. Trends Endocrinol Metab.

[10] Haus E, Smolensky M (2006). Biological clocks and shift work: circadian dysregulation and potential long-term effects. Cancer Causes Control.

[11] Falchi F, Cinzano P, Elvidge CD, Keith DM, Haim A (2011). Limiting the impact of light pollution on human health, environment and stellar visibility. J Environ Manage.

[12] Cinzano P, Falchi F, Elvidge CD (2001). The first World Atlas of the artificial night sky brightness. Mon Not R Astron Soc.

[13] Falchi F, Cinzano P, Duriscoe D, Kyba CC, Elvidge CD, Baugh K (2016). The new world atlas of artificial night sky brightness. Sci Adv.

[14] Falchi F, Bara S (2023). Light pollution is skyrocketing. Science.

[15] Kyba CCM, Kuester T, de SanchezMiguel A, Baugh K, Jechow A, Holker F (2017). Artificially lit surface of Earth at night increasing in radiance and extent. Sci Adv.

[16] Foreman J, Salim AT, Praveen A, Fonseka D, Ting DSW, Guang He M (2021). Association between digital smart device use and myopia: a systematic review and meta-analysis. Lancet Digit Health.

[17] West KE, Jablonski MR, Warfield B, Cecil KS, James M, Ayers MA (2011). Blue light from light-emitting diodes elicits a dose-dependent suppression of melatonin in humans. J Appl Physiol (1985).

[18] Paksarian D, Rudolph KE, Stapp EK, Dunster GP, He J, Mennitt D (2020). Association of outdoor artificial light at night with mental disorders and sleep patterns among US adolescents. JAMA Psychiat.

[19] Wang H, Ma X, Yu Z, Hu N, Du Y, He X (2023). Exposure to outdoor artificial light at night increases risk and burden of metabolic disease in Ningxia, China. Environ Sci Pollut Res Int.

[20] Yu Z, Hu N, Du Y, Wang H, Pu L, Zhang X (2022). Association of outdoor artificial light at night with mental health among China adults: a prospective ecology study. Environ Sci Pollut Res Int.

[21] Lai KY, Sarkar C, Ni MY, Gallacher J, Webster C (2020). Exposure to light at night (LAN) and risk of obesity: a systematic review and meta-analysis of observational studies. Environ Res.

[22] Tancredi S, Urbano T, Vinceti M, Filippini T (2022). Artificial light at night and risk of mental disorders: a systematic review. Sci Total Environ.

[23] Urbano T, Vinceti M, Wise LA, Filippini T (2021). Light at night and risk of breast cancer: a systematic review and dose-response meta-analysis. Int J Health Geogr.

[24] Lei T, Hua H, Du H, Xia J, Xu D, Liu W, et al. Molecular mechanisms of artificial light at night affecting circadian rhythm disturbance. Arch Toxicol. 2023. 10.1007/s00204-023-03647-5 38103071

[25] Nakamura Y, Harama D, Shimokawa N, Hara M, Suzuki R, Tahara Y (2011). Circadian clock gene Period2 regulates a time-of-day-dependent variation in cutaneous anaphylactic reaction. J Allergy Clin Immunol.

[26] Nakao A (2018). Clockwork allergy: how the circadian clock underpins allergic reactions. J Allergy Clin Immunol.

[27] Nakao A (2020). Circadian regulation of the biology of allergic disease: clock disruption can promote allergy. Front Immunol.

[28] Nakao A, Nakamura Y (2022). Time will tell about mast cells: Circadian control of mast cell activation. Allergol Int.

[29] Nakao A, Nakamura Y, Shibata S (2015). The circadian clock functions as a potent regulator of allergic reaction. Allergy.

[30] Durrington HJ, Gioan-Tavernier GO, Maidstone RJ, Krakowiak K, Loudon ASI, Blaikley JF (2018). Time of day affects eosinophil biomarkers in asthma: implications for diagnosis and treatment. Am J Respir Crit Care Med.

[31] Sutherland ER (2005). Nocturnal asthma. J Allergy Clin Immunol.

[32] Cheng FL, An YF, Han ZQ, Li C, Li ZQ, Yang PC (2020). Period2 gene regulates diurnal changes of nasal symptoms in an allergic rhinitis mouse model. Int Forum Allergy Rhinol.

[33] Kim HK, Kim HJ, Kim JH, Kim TH, Lee SH (2018). Asymmetric expression level of clock genes in left vs. right nasal mucosa in humans with and without allergies and in rats: Circadian characteristics and possible contribution to nasal cycle. PLoS ONE.

[34] Matsui MS, Pelle E, Dong K, Pernodet N. Biological Rhythms in the Skin. Int J Mol Sci. 2016;17(6). 10.3390/ijms17060801 PMC4926335 27231897

[35] Loh W, Tang MLK. The Epidemiology of Food Allergy in the Global Context. Int J Environ Res Public Health. 2018;15(9). 10.3390/ijerph15092043 PMC6163515 30231558

[36] Page MJ, McKenzie JE, Bossuyt PM, Boutron I, Hoffmann TC, Mulrow CD (2021). The PRISMA 2020 statement: an updated guideline for reporting systematic reviews. BMJ.

[37] Deprato A, Rao H, Durrington H, Maidstone R, Adan A, Navarro JF, et al. The influence of artificial light at night on asthma and allergy, mental health, and cancer outcomes: a systematic scoping review protocol. Int J Environ Res Public Health. 2022;19(14). 10.3390/ijerph19148522 PMC9319466 35886376

[38] Bland JM, Altman DG (2000). The odds ratio. BMJ.

[39] Huedo-Medina TB, Sanchez-Meca J, Marin-Martinez F, Botella J (2006). Assessing heterogeneity in meta-analysis: Q statistic or I2 index?. Psychol Methods.

[40] Higgins JP, Thompson SG, Deeks JJ, Altman DG (2003). Measuring inconsistency in meta-analyses. BMJ.

[41] ROBINS-E Development Group. Risk Of Bias In Non-randomized Studies - of Exposure (ROBINS-E). [updated Launch version, 1 June 2022. Available from: https://www.riskofbias.info/welcome/robins-e-tool.

[42] Guyatt GH, Oxman AD, Vist GE, Kunz R, Falck-Ytter Y, Alonso-Coello P (2008). GRADE: an emerging consensus on rating quality of evidence and strength of recommendations. BMJ.

[43] Egger M, Davey Smith G, Schneider M, Minder C (1997). Bias in meta-analysis detected by a simple, graphical test. BMJ.

[44] Begg CB, Mazumdar M (1994). Operating characteristics of a rank correlation test for publication bias. Biometrics.

[45] Tang Z, Li S, Shen M, Xiao Y, Su J, Tao J (2022). Association of exposure to artificial light at night with atopic diseases: a cross-sectional study in college students. Int J Hyg Environ Health.

[46] Basnet S, Merikanto I, Lahti T, Mannisto S, Laatikainen T, Vartiainen E (2018). Seasonality, morningness-eveningness, and sleep in common non - communicable medical conditions and chronic diseases in a population. Sleep Sci.

[47] Chen Y, Zhao A, Lyu J, Hu Y, Yin Y, Qu J (2022). Association of delayed chronotype with allergic diseases in primary school children. Chronobiol Int.

[48] Ferreira LGF, Carvalho DAF, Alves FR, Bruin VMS, Bruin PFC (2022). Asthma control, social jetlag, and sleep impairment in high school adolescents. Sleep Med.

[49] Haldar P, Carsin AE, Debnath S, Maity SG, Annesi-Maesano I, Garcia-Aymerich J, et al. Individual circadian preference (chronotype) is associated with asthma and allergic symptoms among adolescents. ERJ Open Res. 2020;6(2). 10.1183/23120541.00226-2020 PMC7335840 32665950

[50] Han CH, Chung J. Late Chronotype is Associated with Adolescent Asthma: Assessment Using the Korean-Version MCTQ. Int J Environ Res Public Health. 2020;17(9). 10.3390/ijerph17093000 PMC7246473 32357431

[51] Merikanto I, Englund A, Kronholm E, Laatikainen T, Peltonen M, Vartiainen E (2014). Evening chronotypes have the increased odds for bronchial asthma and nocturnal asthma. Chronobiol Int.

[52] Fischer FM, Morata TC, LatorreMdo R, Krieg EF, Fiorini AC, Colacioppo S (2001). Effects of environmental and organizational factors on the health of shiftworkers of a printing company. J Occup Environ Med.

[53] Huang Y, Jing D, Su J, Huang Z, Liu H, Tao J (2021). Association of night shift work with chronic spontaneous urticaria and effect modification by circadian dysfunction among workers. Front Public Health.

[54] Kim B, Jung H, Kim J, Lee J, Kim O. Depressive symptoms and sleep disturbance in female nurses with atopic dermatitis: the Korea nurses' health study. Int J Environ Res Public Health. 2020;17(8). 10.3390/ijerph17082743 PMC7215983 32316146

[55] Lu F, Suggs A, Ezaldein HH, Ya J, Fu P, Jamora J (2019). The effect of shift work and poor sleep on self-reported skin conditions: a survey of call center agents in the Philippines. Clocks Sleep.

[56] Maidstone RJ, Turner J, Vetter C, Dashti HS, Saxena R, Scheer F (2021). Night shift work is associated with an increased risk of asthma. Thorax.

[57] Chang YS, Chiang BL (2016). Mechanism of sleep disturbance in children with atopic dermatitis and the role of the circadian rhythm and melatonin. Int J Mol Sci.

[58] Czeisler CA, Shanahan TL, Klerman EB, Martens H, Brotman DJ, Emens JS (1995). Suppression of melatonin secretion in some blind patients by exposure to bright light. N Engl J Med.

[59] Fishbein AB, Knutson KL, Zee PC. Circadian disruption and human health. J Clin Invest. 2021;131(19). 10.1172/JCI148286 PMC8483747 34596053

[60] Stevens RG, Zhu Y. Electric light, particularly at night, disrupts human circadian rhythmicity: is that a problem? Philos Trans R Soc Lond B Biol Sci. 2015;370(1667). 10.1098/rstb.2014.0120 PMC4375361 25780233

[61] Reinberg A, Ghata J, Sidi E (1963). Nocturnal asthma attacks: their relationship to the circadian adrenal cycle. J Allergy.

[62] Greenberg H, Cohen RI (2012). Nocturnal asthma. Curr Opin Pulm Med.

[63] Turek FW (2016). Circadian clocks: not your grandfather's clock. Science.

[64] Roenneberg T, Kuehnle T, Juda M, Kantermann T, Allebrandt K, Gordijn M (2007). Epidemiology of the human circadian clock. Sleep Med Rev.

[65] Duffy JF, Zitting KM, Chinoy ED (2015). Aging and circadian rhythms. Sleep Med Clin.

[66] Shawa N, Roden LC (2016). Chronotype of South African adults is affected by solar entrainment. Chronobiol Int.

[67] Randler C, Prokop P, Sahu S, Haldar P (2015). Cross-cultural comparison of seven morningness and sleep-wake measures from Germany. India and Slovakia Int J Psychol.

[68] GBD. GBD 2019 Chronic Respiratory Diseases Collaborators. Prevalence and attributable health burden of chronic respiratory diseases, 1990–2017: a systematic analysis for the Global Burden of Disease Study 2017. Lancet Respir Med. 2020;8(6):585–96. 10.1016/S2213-2600(20)30105-3 PMC7284317 32526187

[69] Paganelli R, Petrarca C, Di Gioacchino M (2018). Biological clocks: their relevance to immune-allergic diseases. Clin Mol Allergy.

[70] Yang H, Yang LT, Liu J, Tang S, Zhao X, Wang Q (2018). Circadian protein CLK suppresses transforming growth factor-beta expression in peripheral B cells of nurses with day-night shift rotation. Am J Transl Res.

[71] Wang Q, Li L, Li C, Cao H, Chen Y, Zhou W (2022). Circadian protein CLOCK modulates regulatory B cell functions of nurses engaging day-night shift rotation. Cell Signal.

[72] Durrington HJ, Krakowiak K, Meijer P, Begley N, Maidstone R, Goosey L, et al. Circadian asthma airway responses are gated by REV-ERBalpha. Eur Respir J. 2020;56(6). 10.1183/13993003.02407-2019 PMC7613655 32586876

[73] Smolensky MH, Lemmer B, Reinberg AE (2007). Chronobiology and chronotherapy of allergic rhinitis and bronchial asthma. Adv Drug Deliv Rev.

[74] Spengler CM, Shea SA (2000). Endogenous circadian rhythm of pulmonary function in healthy humans. Am J Respir Crit Care Med.

[75] Wilkinson M, Maidstone R, Loudon A, Blaikley J, White IR, Singh D, et al. Circadian rhythm of exhaled biomarkers in health and asthma. Eur Respir J. 2019;54(4). 10.1183/13993003.01068-2019 PMC6796150 31221808

[76] Cheng FL, An YF, Xue JM, Wang YJ, Ding XW, Zhang YT (2022). Circadian rhythm disruption exacerbates Th2-like immune response in murine allergic airway inflammation. Int Forum Allergy Rhinol.

[77] Harding BN, Aguilar R, Espinosa A, Castano-Vinyals G, Papantoniou K, Navarrete JM (2022). Disruption of cellular immune response among male rotating night shift workers in Spain- The HORMONIT study. Front Immunol.

[78] Marseglia L, D'Angelo G, Manti S, Salpietro C, Arrigo T, Barberi I (2014). Melatonin and atopy: role in atopic dermatitis and asthma. Int J Mol Sci.

[79] Le Fur I, Reinberg A, Lopez S, Morizot F, Mechkouri M, Tschachler E (2001). Analysis of circadian and ultradian rhythms of skin surface properties of face and forearm of healthy women. J Invest Dermatol.

[80] Dimitrov S, Lange T, Nohroudi K, Born J (2007). Number and function of circulating human antigen presenting cells regulated by sleep. Sleep.

[81] Vaughn AR, Clark AK, Sivamani RK, Shi VY (2018). Circadian rhythm in atopic dermatitis-Pathophysiology and implications for chronotherapy. Pediatr Dermatol.

[82] Sherman H, Froy O (2008). Expression of human beta-defensin 1 is regulated via c-Myc and the biological clock. Mol Immunol.

[83] Chang YS, Chou YT, Lee JH, Lee PL, Dai YS, Sun C (2014). Atopic dermatitis, melatonin, and sleep disturbance. Pediatrics.

[84] Arshad SH, Karmaus W, Raza A, Kurukulaaratchy RJ, Matthews SM, Holloway JW (2012). The effect of parental allergy on childhood allergic diseases depends on the sex of the child. J Allergy Clin Immunol.

[85] de Benedictis FM, Attanasi M (2016). Asthma in childhood. Eur Respir Rev.

[86] Milligan KL, Matsui E, Sharma H (2016). Asthma in urban children: epidemiology, environmental risk factors, and the public health domain. Curr Allergy Asthma Rep.

[87] Vaughn AR, Sivamani RK, Lio PA, Shi VY (2017). Paternal vs. maternal factors in childhood atopic dermatitis. Dermatitis.

[88] Khreis H, Kelly C, Tate J, Parslow R, Lucas K, Nieuwenhuijsen M (2017). Exposure to traffic-related air pollution and risk of development of childhood asthma: a systematic review and meta-analysis. Environ Int.

[89] Eguiluz-Gracia I, Mathioudakis AG, Bartel S, Vijverberg SJH, Fuertes E, Comberiati P (2020). The need for clean air: the way air pollution and climate change affect allergic rhinitis and asthma. Allergy.

[90] Qiu AY, Leng S, McCormack M, Peden DB, Sood A (2022). Lung effects of household air pollution. J Allergy Clin Immunol Pract.

[91] Paciencia I, Cavaleiro Rufo J, Moreira A. Environmental inequality: Air pollution and asthma in children. Pediatr Allergy Immunol. 2022;33(6). 10.1111/pai.13818 35754123

[92] Nanda A, Mustafa SS, Castillo M, Bernstein JA (2022). Air pollution effects in allergies and asthma. Immunol Allergy Clin North Am.

