# Supplementary material for: Influence of light at night on allergic diseases: a systematic review and meta-analysis
Source: BMC Med. 2024 Feb 14;22:67. doi: 10.1186/s12916-024-03291-5 (PMC10865638; doi:10.1186/s12916-024-03291-5)
Supplement: Supplementary file 1 — Additional file 1: Table S1. Comprehensive strategy of the initial search to identify studies considering the influence of light at night exposure on allergic diseases. Table S2. Comprehensive strategy of the updated search to identify studies considering the influence of light at night exposure on allergic diseases. Table S3. Summary of meta-analyses considering the association between light at night exposure and the odds of allergic diseases. Fig. S1. Funnel plot of the exposure-specific meta-analysis for the association between light at night exposure and the odds of allergic diseases. Fig. S2. Funnel plot of the outcome-specific meta-analysis for the association between light at night exposure and the odds of allergic diseases. Fig. S3. Risk of Bias In Non-randomized Studies – of Exposures (ROBINS-E) quality assessment of included studies considering the influence of light at night exposure on allergic diseases. Table S4. Grading of Recommendations, Assessment, Development, and Evaluation (GRADE) criteria evidence table of meta-analyses considering the influence of light at night exposure on the odds of allergic diseases. [file 12916_2024_3291_MOESM1_ESM.docx]

# **Supplementary Material**

# **Influence of Light at Night on Allergic Diseases: A Systematic Review and Meta-Analysis**

Andy Deprato BScKin,^1,2^ Robert Maidstone PhD,^3^ Anna Palomar Cros PhD,^4,5,6^ Ana Adan PhD,^7,8^ Prasun Haldar PhD,^9^ Barbara N Harding PhD,^4,5,6^ Paige Lacy PhD,^1^ Lyle Melenka MD, MSc,^10^ Saibal Moitra MD, PhD,^11^ José Francisco Navarro PhD,^12^ Manolis Kogevinas MD, PhD,^4,5,6,13^ Hannah Durrington MD, PhD,^3^ Subhabrata Moitra PhD^1,14^

1. Alberta Respiratory Centre and Division of Pulmonary Medicine, University of Alberta, Edmonton, Alberta, Canada
2. Michael G. DeGroote School of Medicine, McMaster University, Hamilton, Ontario, Canada
3. Division of Immunology, Immunity to Infection, and Respiratory Medicine, University of Manchester, Manchester, United Kingdom
4. Non-Communicable Diseases and Environment Programme, Barcelona Institute for Global Health (ISGlobal), Barcelona, Spain
5. Department of Experimental and Health Sciences, University of Pompeu Fabra (UPF), Barcelona, Spain
6. Consortium for Biomedical Research in Epidemiology and Public Health (CIBERESP), Carlos III Institute of Health, Madrid, Spain
7. Department of Clinical Psychology and Psychobiology, University of Barcelona, Barcelona, Spain
8. Institute of Neurosciences, University of Barcelona, Barcelona, Spain
9. Department of Medical Laboratory Technology, Supreme Institute of Management and Technology, Mankundu, India
10. Synergy Respiratory and Cardiac Care, Sherwood Park, Alberta, Canada
11. Department of Allergy and Immunology, Apollo Gleneagles Hospital, Kolkata, India
12. Department of Psychobiology and Methodology of Behavioural Sciences, University of Málaga, Málaga, Spain
13. Hospital del Mar Medical Research Institute (IMIM), Barcelona, Spain
14. Canadian VIGOUR Centre, Department of Medicine, University of Alberta, Edmonton, Alberta, Canada

**Correspondence to:**

Dr. Subhabrata Moitra, Alberta Respiratory Centre and Division of Pulmonary Medicine, 574B Heritage Medical Research Centre, University of Alberta, Edmonton, Alberta, Canada T6G 2S2

Tel: 1.780.604.0263

Email: moitra@ualberta.ca

**Table S1** Comprehensive strategy of the initial search to identify studies considering the influence of light at night exposure on allergic diseases.

| **Database** | **Search Strategy** | **Records** |
| --- | --- | --- |
| MEDLINE (1946 - present via OVID) | Ovid MEDLINE(R) ALL <1946 to December 02, 2022>   1. (artificial light at night or ALAN or light at night or light pollution or chronotype or shift work or shiftwork).mp. 10819 2. (asthma* or allerg* or immune).mp. 1222580 3. 1 and 2 251   mp = title, book title, abstract, original title, name of substance word, subject heading word, floating sub-heading word, keyword heading word, organism supplementary concept word, protocol supplementary concept word, rare disease supplementary concept word, unique identifier, synonyms | 251 |
| EMBASE (1974 - present via OVID) | Embase <1974 to 2022 December 02>   1. (artificial light at night or ALAN or light at night or light pollution or chronotype or shift work or shiftwork).mp. 15201 2. (asthma* or allerg* or immune).mp. 1923567 3. 1 and 2 427   mp = title, abstract, heading word, drug trade name, original title, device manufacturer, drug manufacturer, device trade name, keyword heading word, floating subheading word, candidate term word | 427 |
| PubMed (with links to UofA e-journals) | All Fields: ("artificial light at night" OR ALAN OR "light at night" OR "light pollution" OR "chronotype" OR "shift work" OR shiftwork) AND (asthma* OR allerg* OR immune) | 912 |
| Scopus | ( TITLE-ABS-KEY ( "artificial light at night" OR alan OR "light at night" OR "light pollution" OR "chronotype" OR "shift work" OR shiftwork ) AND TITLE-ABS-KEY ( asthma* OR allerg* OR immune ) ) | 572 |
| Web of Science Core Collection | 1. TS = ("artificial light at night" OR ALAN OR "light at night" OR "light pollution" OR "chronotype" OR "shift work" OR shiftwork)   Date Run: Sat Dec 03 2022 16:00:34 GMT-0700 (Mountain Standard Time)  Results: 19444   1. TS = (asthma* OR allerg* OR immune)   Date Run: Sat Dec 03 2022 16:01:01 GMT-0700 (Mountain Standard Time)  Results: 1272651   1. #1 AND #2   Date Run: Sat Dec 03 2022 16:01:09 GMT-0700 (Mountain Standard Time)  Results: 347 | 347 |
| Cochrane Reviews (Cochrane Library) | 1. "artificial light at night" OR ALAN OR "light at night" OR "light pollution" OR "chronotype" OR "shift work" OR shiftwork 1316 2. asthma* OR allerg* OR immune 104955 3. #1 AND #2 93 | 63 |
| Cochrane Protocols (Cochrane Library) | 1. "artificial light at night" OR ALAN OR "light at night" OR "light pollution" OR "chronotype" OR "shift work" OR shiftwork 1316 2. asthma* OR allerg* OR immune 104955 3. #1 AND #2 93 | 3 |
| International Prospective Register of Systematic Reviews (PROSPERO) | ("artificial light at night" OR ALAN OR "light at night" OR "light pollution" OR "chronotype" OR "shift work" OR shiftwork) AND (asthma* OR allerg* OR immune) | 67 |
| Google Scholar | ("artificial light at night" OR ALAN OR "light at night" OR "light pollution" OR "chronotype" OR "shift work" OR shiftwork) AND (asthma* OR allerg* OR immune) | First 100 results used |

**Table S2** Comprehensive strategy of the updated search to identify studies considering the influence of light at night exposure on allergic diseases.

| **Database** | **Search Strategy** | **Results** |
| --- | --- | --- |
| MEDLINE (1946 - present via OVID) | Ovid MEDLINE(R) ALL <1946 to December 07, 2023>   1. (artificial light at night or ALAN or light at night or light pollution or chronotype or shift work or shiftwork).mp. 11931 2. (asthma* or allerg* or immune).mp. 1308981 3. 1 and 2 274 4. limit 3 to yr="2022 -Current" 63   mp = title, book title, abstract, original title, name of substance word, subject heading word, floating sub-heading word, keyword heading word, organism supplementary concept word, protocol supplementary concept word, rare disease supplementary concept word, unique identifier, synonyms, population supplementary concept word, anatomy supplementary concept word | 63 |
| EMBASE (1974 - present via OVID) | Embase <1974 to 2023 December 13>   1. (artificial light at night or ALAN or light at night or light pollution or chronotype or shift work or shiftwork).mp. 16775 2. (asthma* or allerg* or immune).mp. 2077529 3. 1 and 2 484 4. limit 3 to yr="2022 -Current" 104   mp = title, abstract, heading word, drug trade name, original title, device manufacturer, drug manufacturer, device trade name, keyword heading word, floating subheading word, candidate term word | 104 |
| PubMed | ("artificial light at night" OR ALAN OR "light at night" OR "light pollution" OR "chronotype" OR "shift work" OR shiftwork) AND (asthma* OR allerg* OR immune)  Results by Year: 2022-2024 | 209 |
| Scopus | ( TITLE-ABS-KEY ( "artificial light at night" OR alan OR "light at night" OR "light pollution" OR "chronotype" OR "shift work" OR shiftwork ) AND TITLE-ABS-KEY ( asthma* OR allerg* OR immune ) ) AND PUBYEAR > 2021 | 95 |
| Web of Science Core Collection | 1. TS=("artificial light at night" OR ALAN OR "light at night" OR "light pollution" OR "chronotype" OR "shift work" OR shiftwork)   Date Run: Fri Dec 15 2023 18:42:37 GMT-0500 (Eastern Standard Time)  Results: 21131   1. TS=(asthma* OR allerg* OR immune)   Date Run: Fri Dec 15 2023 18:42:55 GMT-0500 (Eastern Standard Time)  Results: 1358086   1. #1 AND #2   Date Run: Fri Dec 15 2023 18:43:00 GMT-0500 (Eastern Standard Time)  Results: 392   1. #3 Timespan: 2022-12-03 to 2023-12-16   Date Run: Fri Dec 15 2023 18:44:33 GMT-0500 (Eastern Standard Time)  Results: 41 | 41 |
| Cochrane Reviews (Cochrane Library) | 1. "artificial light at night" OR ALAN OR "light at night" OR "light pollution" OR "chronotype" OR "shift work" OR shiftwork 1410 2. asthma* OR allerg* OR immune 111207 3. #1 AND #2 98 4. #3 with Cochrane Library publication date Between Dec 2022 and Dec 2023 5 | 3 |
| Cochrane Protocols (Cochrane Library) | 1. "artificial light at night" OR ALAN OR "light at night" OR "light pollution" OR "chronotype" OR "shift work" OR shiftwork 1410 2. asthma* OR allerg* OR immune 111207 3. #1 AND #2 98 4. #3 with Cochrane Library publication date Between Dec 2022 and Dec 2023 5 | No results returned |
| International Prospective Register of Systematic Reviews (PROSPERO) | ("artificial light at night" OR ALAN OR "light at night" OR "light pollution" OR "chronotype" OR "shift work" OR shiftwork) AND (asthma* OR allerg* OR immune) WHERE CD FROM 03/12/2022 TO 15/12/2023 | 11 |
| Google Scholar | ("artificial light at night" OR ALAN OR "light at night" OR "light pollution" OR "chronotype" OR "shift work" OR shiftwork) AND (asthma* OR allerg* OR immune)  Publication Date: Since 2022 | First 100 results used |

**Table S3** Summary of meta-analyses considering the association between light at night exposure and the odds of allergic diseases.

| **Outcome** | **Comparison** | **Studies (n)** | **OR (95% CI)** | **p-value** | **I^2^** | **Certainty** |
| --- | --- | --- | --- | --- | --- | --- |
| Exposure stratification | Artificial light at night | 1 (20 106) | 1.88 (1.04, 3.39) | p = 0.04 | N/A | N/A |
|  | Chronotype | 6 (304 493) | 1.35 (0.98, 1.87) | p = 0.07 | 91% | Very low |
|  | Shift work | 5 (531 318) | 1.33 (1.06, 1.67) | p = 0.02 | 74% | Low |
| Outcome stratification | Asthma | 9 (827 263) | 1.62 (1.19, 2.20) | p = 0.002 | 93% | Very low |
|  | Allergic rhinitis | 5 (38 412) | 1.89 (1.60, 2.24) | p < 0.0001 | 59% | Moderate |
|  | Skin allergies | 7 (60 977) | 1.44 (1.09, 1.91) | p = 0.01 | 89% | Very low |
| Artificial light at night stratification | Asthma | 1 (20 106) | 3.51 (2.56, 4.82) | p < 0.00001 | N/A | N/A |
|  | Allergic rhinitis | 1 (20 106) | 1.97 (1.77, 2.20) | p < 0.00001 | N/A | N/A |
|  | Skin allergies | 1 (20 106) | 1.00 (0.85, 1.18) | p = 0.97 | N/A | N/A |
| Chronotype stratification | Asthma | 6 (304 493) | 1.49 (1.02, 2.17) | p = 0.04 | 92% | N/A |
|  | Allergic rhinitis | 3 (18 182) | 1.86 (1.41, 2.46) | p < 0.0001 | 72% | N/A |
|  | Skin allergies | 2 (12 093) | 1.84 (1.20, 2.83) | p = 0.005 | 70% | N/A |
| Shift work stratification | Asthma | 2 (502 664) | 1.22 (1.02, 1.44) | p = 0.03 | 0% | N/A |
|  | Allergic rhinitis | 1 (124) | 2.79 (1.18, 6.60) | p = 0.02 | N/A | N/A |
|  | Skin allergies | 4 (28 778) | 1.42 (0.96, 2.09) | p = 0.08 | 76% | N/A |
| Age stratification | Youth | 4 (291 980) | 1.63 (1.07, 2.48) | p = 0.02 | 90% | N/A |
|  | Adults | 8 (563 937) | 1.30 (1.03, 1.63) | p = 0.02 | 81% | N/A |
| Region stratification | Americas | 2 (1581) | 2.35 (1.40, 3.96) | p = 0.001 | 33% | N/A |
|  | Asia | 7 (339 283) | 1.35 (1.13, 1.61) | p = 0.0009 | 84% | N/A |
|  | Europe | 3 (515 053) | 1.04 (0.66, 1.65) | p = 0.86 | 89% | N/A |

OR: odds ratio; 95% CI: 95% confidence interval

**Fig. S1** Funnel plot of the exposure-specific meta-analysis for the association between light at night exposure and the odds of allergic diseases.


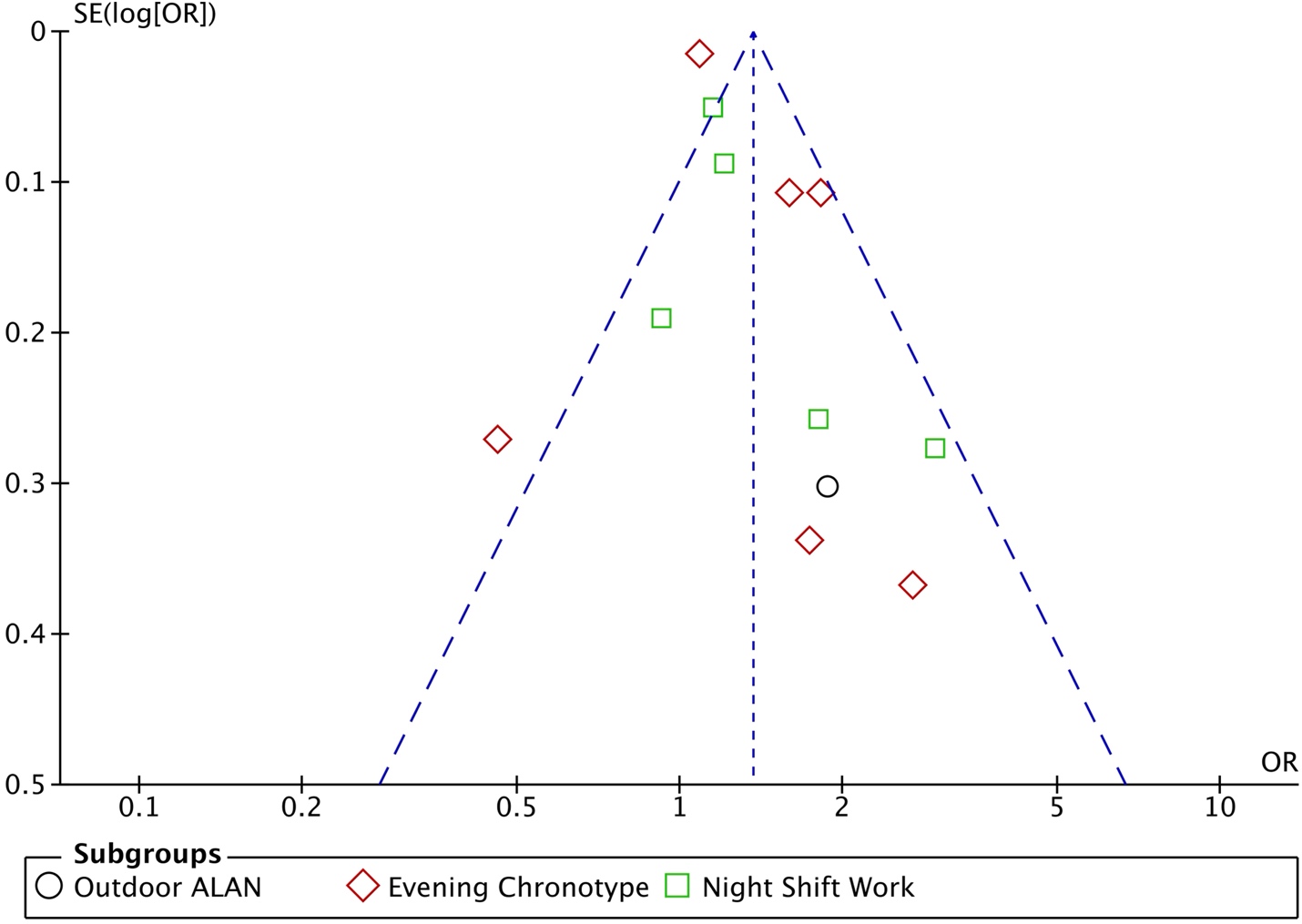


For chronotype, Egger’s test p-value: 0.31 and Begg’s test p-value: 0.85. For shift work, Egger’s test p-value: 0.27 and Begg’s test p-value: 0.14. Bias could not be computed for outdoor ALAN (n=1).

**Fig. S2** Funnel plot of the outcome-specific meta-analysis for the association between light at night exposure and the odds of allergic diseases.


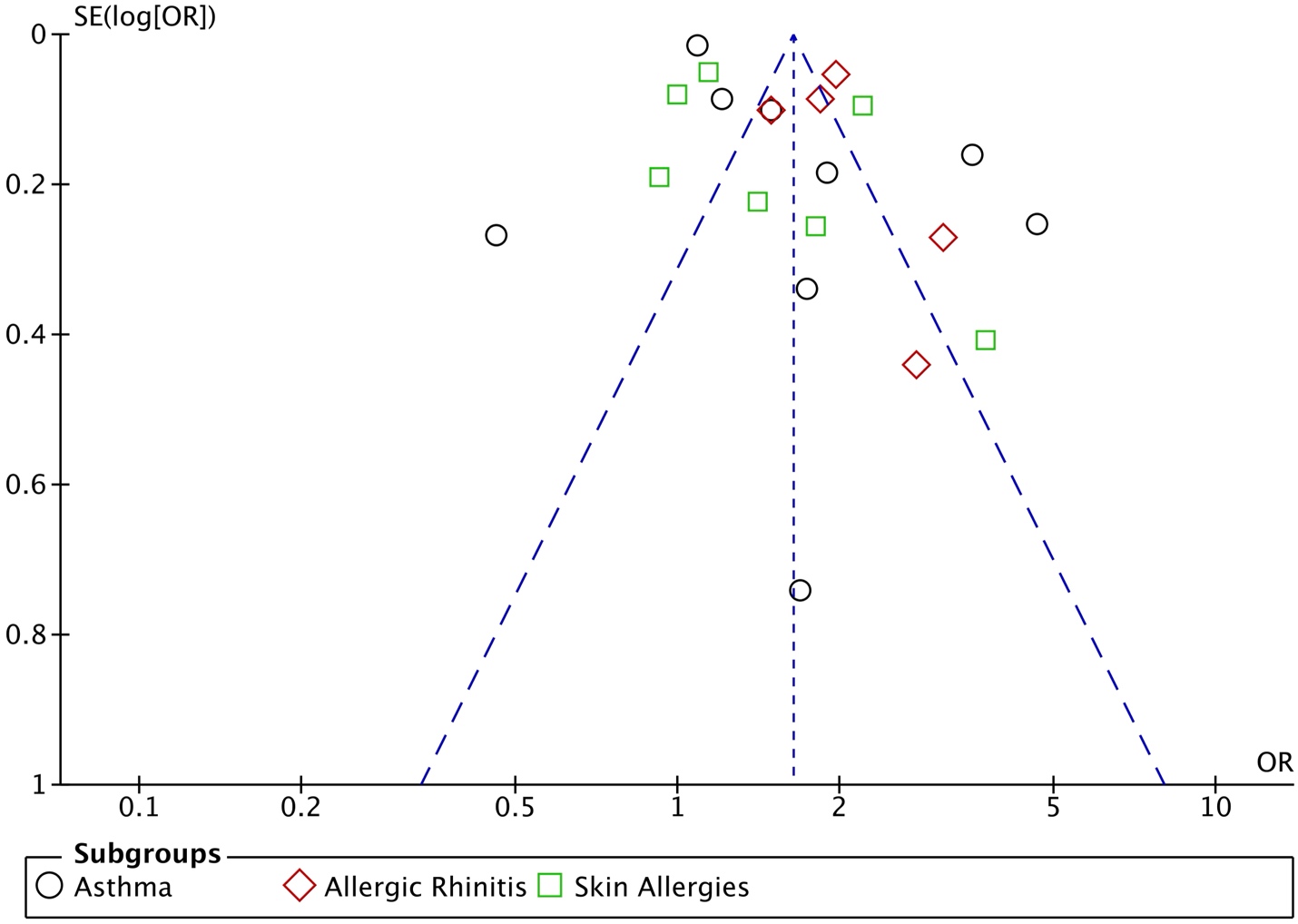


For asthma, Egger’s test p-value: 0.09 and Begg’s test p-value: 1.00. For allergic rhinitis, Egger’s test p-value: 0.17 and Begg’s test p-value: 0.14. For skin allergies, Egger’s test p-value: 0.26 and Begg’s test p-value: 0.19.

**Fig. S3** Risk of Bias In Non-randomized Studies – of Exposures (ROBINS-E) quality assessment of included studies considering the influence of light at night exposure on allergic diseases.


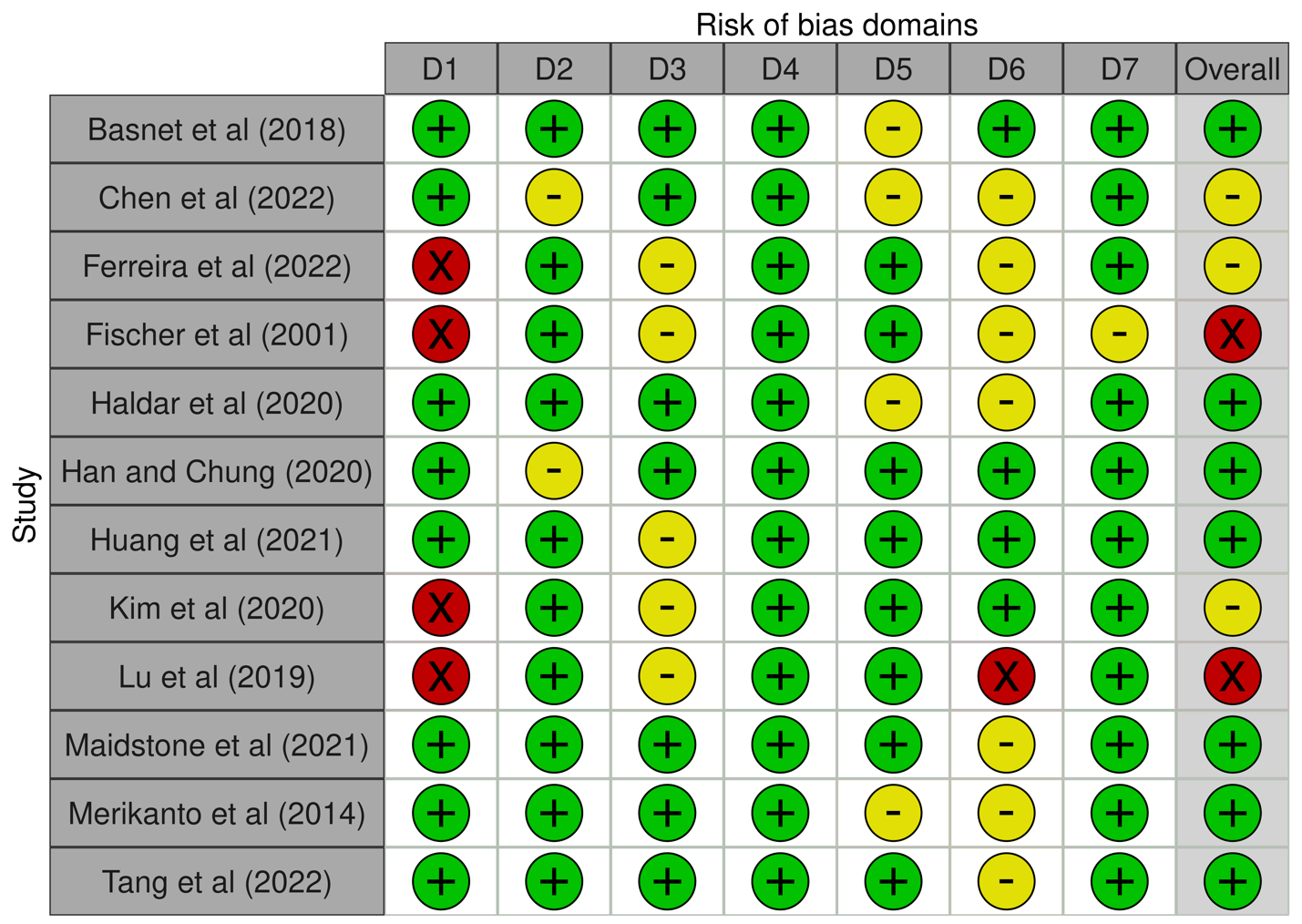


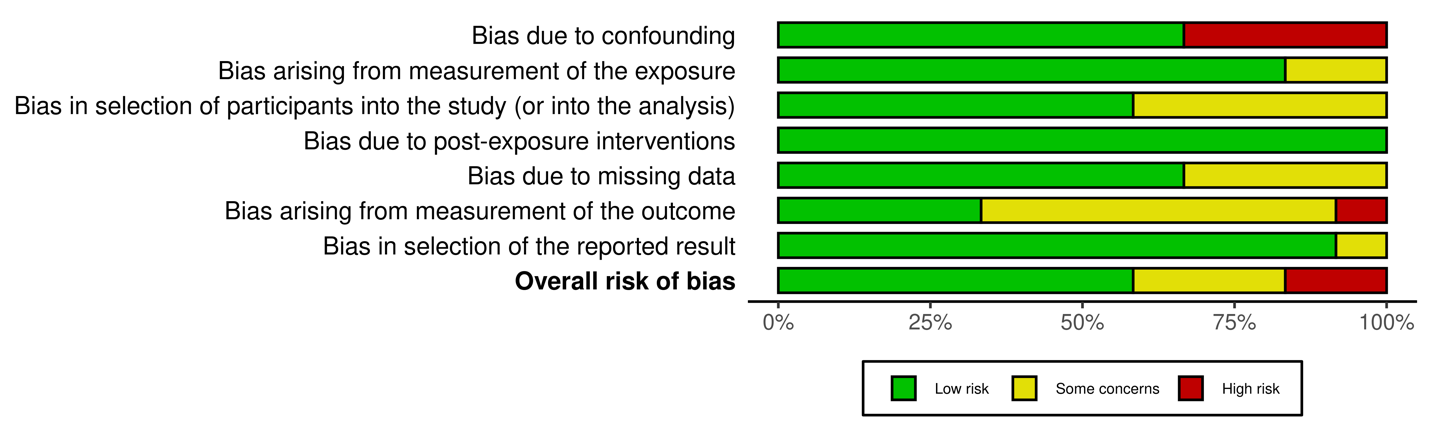


(1-12)

**Table S4** Grading of Recommendations, Assessment, Development, and Evaluation (GRADE) criteria evidence table of meta-analyses considering the influence of light at night exposure on the odds of allergic diseases.

| **Certainty assessment** | | | | | | | **№ of patients** | | **Effect** | | **Certainty** | **Importance** |
| --- | --- | --- | --- | --- | --- | --- | --- | --- | --- | --- | --- | --- |
| **№ of studies** | **Study design** | **Risk of bias** | **Inconsistency** | **Indirectness** | **Imprecision** | **Other considerations** | **Asthma and Allergic Diseases** | **placebo** | **Relative (95% CI)** | **Absolute (95% CI)** |  |  |
| **Outdoor ALAN** | | | | | | | | | | | | |
| 1 | N/A | N/A | N/A | N/A | N/A | N/A | N/A | N/A | **OR 1.88** (1.04 to 3.39) | **2 fewer per 1,000** (from 3 fewer to 1 fewer) | N/A | N/A |
| **Evening Chronotype** | | | | | | | | | | | | |
| 6 | non-randomised studies | not serious | serious | not serious | not serious | none | N/A | N/A | **OR 1.35** (0.98 to 1.87) | **1 fewer per 1,000** (from 2 fewer to 1 fewer) | ⨁◯◯◯ Very low | N/A |
| **Night Shift Work** | | | | | | | | | | | | |
| 5 | non-randomised studies | not serious | not serious | not serious | not serious | none | N/A | N/A | **OR 1.33** (1.06 to 1.67) | **1 fewer per 1,000** (from 2 fewer to 1 fewer) | ⨁⨁◯◯ Low | N/A |
| **Asthma** | | | | | | | | | | | | |
| 9 | non-randomised studies | not serious | serious | not serious | not serious | none | N/A | N/A | **OR 1.62** (1.19 to 2.20) | **2 fewer per 1,000** (from 2 fewer to 1 fewer) | ⨁◯◯◯ Very low | N/A |
| **Allergic Rhinitis** | | | | | | | | | | | | |
| 5 | non-randomised studies | not serious | not serious | not serious | not serious | strong association | N/A | N/A | **OR 1.89** (1.60 to 2.24) | **2 fewer per 1,000** (from 2 fewer to 2 fewer) | ⨁⨁⨁◯ Moderate | N/A |
| **Skin Allergies** | | | | | | | | | | | | |
| 7 | non-randomised studies | not serious | serious | not serious | not serious | none | N/A | N/A | **OR 1.44** (1.09 to 1.91) | **1 fewer per 1,000** (from 2 fewer to 1 fewer) | ⨁◯◯◯ Very low | N/A |
